# Dural ectopic lymphatic structures accumulate during aging and exhibit dysregulation in neurodegenerative diseases

**DOI:** 10.1073/pnas.2425081122

**Published:** 2025-08-12

**Authors:** Amit Fruitman Davidi, Sophie Shirenova, Dolev Michaelovich, Giulio Benedetti, Hadar Shtrasberg, Irit Shoval, Hagit Hauschner, Gal Oz, Karin Vardy, Tamir Hirsh, Ravit Madar, Edward A. Stern, Hanna Rosenmann, Arya Biragyn, Eitan Okun

**Affiliations:** ^a^The Mina and Everard Goodman, Faculty of Life Sciences, Bar-Ilan University, Ramat Gan 5290002, Israel; ^b^The Leslie and Susan Gonda Multidisciplinary Brain Research Center, Bar-Ilan University, Ramat Gan 5290002, Israel; ^c^The Paul Feder Laboratory on Alzheimer’s Disease Research, Bar-Ilan University, Ramat Gan 5290002, Israel; ^d^Department of Neurology, The Agnes Ginges Center for Human Neurogenetics, Hadassah Hebrew University Medical Center, Jerusalem 9112001, Israel; ^e^Faculty of Medicine, Hebrew University of Jerusalem, Jerusalem 9112102, Israel; ^f^Hadassah BrainLabs-National Knowledge Center for Research on Brain Diseases, Hadassah-Hebrew University Medical Center, Jerusalem 9112001, Israel; ^g^Immunoregulation Section, Laboratory of Immunology and Molecular Biology, National Institute on Aging, Baltimore, MD 21224

**Keywords:** meninges, Alzheimer’s disease, ectopic lymphoid structures, amyloid-beta, Down syndrome

## Abstract

The meninges serve as a critical interface between the peripheral immune system and the central nervous system, playing a crucial role in maintaining parenchymal homeostasis. Neurodegenerative disorders, such as amyloidosis and tauopathies, are marked by the accumulation of extracellular neurotoxic amyloid-β (Aβ) plaques and intracellular tau tangles, respectively, leading to neuronal cell death and cognitive decline. The role of the adaptive immune response in these pathologies remains under debate. Adaptive immune cells can manifest as ectopic lymphoid structures (ELS), which resemble secondary lymphoid organs, and form at sites of inflammation or pathology. While ELSs can support immune responses against infections or tumors, they may also have detrimental effects in certain pathological conditions. To explore whether meningeal ELS are implicated in aging and neurodegeneration, we analyzed the meninges of aged wild-type mice and mouse models of early-onset Alzheimer’s disease, tauopathy, and Down syndrome-related neurodegenerative disorders. Our findings suggest that the accumulation of dural ELS varies according to age, brain pathology, and sex. Meningeal myeloid cells may contribute to the initiation and maintenance of ELS during aging. These results demonstrate the potential contribution of meningeal ELS to healthy aging and neurodegenerative conditions, offering directions for future research.

## Significance

The dynamics of meningeal adaptive immunity, a crucial gateway to the brain, remain unclear. Here, we provide a comprehensive mapping of dural ectopic lymphoid structures (ELS), which exhibit age-, sex-, and brain pathology–specific patterns. Alzheimer’s disease mouse models display distinct ELS trajectories. In 5xFAD mice, ELS expand prematurely and robustly, while APP/PS1 animals show increased structural complexity alongside an age-related decline in ELS. Tauopathy and Down syndrome models exhibit markedly reduced ELS. Meningeal myeloid cells may promote ELS via lymphotoxin-β receptor signaling, suggesting a stromal–immune feedback mechanism. These findings highlight ELS as dynamic indicators of brain pathology, offering a sensitive biomarker and a potential target for precision immunotherapies to combat age-related neurodegeneration.

The meninges, a tissue composed of three distinct layers, namely, the dura mater, the arachnoid space, and the pia mater (1, 2), mediates the interaction between the central nervous system (CNS) and the periphery. It serves as a vital interface for neuroimmune processes and the influence of peripheral immune activity on the brain parenchyma (3). Within the dura mater, B cells constitute approximately 30% of the immune cell population (4), offering a robust presence of the adaptive immune response toward potential infiltration of pathogens into the CNS (5).

Immunity becomes particularly relevant for age-related neurodegenerative conditions such as Alzheimer’s disease (AD) (6) and AD in individuals with Down syndrome (DS) (7). These conditions are characterized by extracellular accumulation of amyloid-β (Aβ) plaques and intracellular tau tangles, causing brain inflammation and neuronal death (8). Age and pathology-related decreased meningeal lymphatic drainage exacerbate Aβ deposition in the brain and worsen microglial function (9, 10). Further, aging impairs the function of the meningeal lymphatics (11), which in turn can contribute to brain function decline. These changes affect lymphatic drainage and may also influence immune cell infiltration and activation within the CNS (12), including CD8^+^ T cells, that infiltrate the hippocampus in an age and pathology-dependent manner (13). In AD patients, B cells were found to secrete immunoglobulin(Ig)G, which can promote Aβ phagocytosis (14), implicating these cells in disease pathology. A number of studies have assessed the impact of adaptive immunity on disease progression in several early-onset AD (EOAD) transgenic (Tg) mouse models, which overexpress the Amyloid precursor protein (APP) and presenilin 1 (PS1) proteins. However, these studies prompted conflicting results (15–17). In one study, permanent or transient B cell depletion in the 3xTgAD and APP/PS1 models reduced Aβ deposition in the brain and improved cognitive function (15), suggesting that B cells exert adverse effects in EOAD. In contrast, immune-compromised mice of the 5x familial AD (5xFAD) mouse model of EOAD, lacking mature B and T cells, exhibit accelerated Aβ accumulation (16), suggesting a beneficial role for adaptive immunity in EOAD. These findings suggest a complex interplay between B cells, Igs, microglial function, and Aβ accumulation in AD pathology, though the underlying mechanism remains unclear. Therefore, the precise role of adaptive immunity in AD, and B cells in particular, is still not fully understood.

Recently, dural-associated lymphatic tissue was identified at the rostro-rhinal sagittal sinus in the dura mater. This specialized immune hub is intricately connected with numerous blood and lymphatic vessels, housing immune aggregates that expand with age. It can acquire and respond to both local and systemic antigens, limiting parenchymal viral load. The presence of germinal centers (GC) and GC-associated B cells in this area suggests that this tissue provides protective roles for the CNS from pathogens (18). Under homeostatic conditions, the meninges are predominantly guarded by naive B cells (IgD^+^ and IgM^+^) as well as IgA^+^ plasma cells derived from gut-associated lymphoid tissue (5). However, this study showed that blood- or rhinal-derived microbial infections activate IgG^+^ B cells and promote immune hub aggregation (18).

Under conditions of persistent inflammation, various tissues acquire characteristics akin to those found in lymph nodes (19–21). Specifically, stromal cells, macrophages, and dendritic cells (DCs) express Lymphotoxin-β receptor (LTβR), which binds to its ligand LTβ. This ligand is expressed on lymphoid tissue inducer cells, which may include B and T cells. This interaction prompts the development of immune aggregates through the secretion of chemokines (C-X-C motif), such as chemokine ligand 13 (CXCL13). These aggregates are also termed immune foci for small follicles and GC-like structures for larger and more complex aggregates (22). In the context of this study, we refer to these adaptive immune cell follicles as ectopic lymphoid structures (ELS). ELSs exhibit a spectrum of cellular compositions, ranging from loose clusters of T and B cells to intricately organized entities featuring distinct T cell zones and B cell areas housing GCs (23, 24). These structures are characterized by their nonencapsulated nature and variable levels of complexity. Indeed, these structures may span from basic clusters with rudimentary segregation of B and T cells to more complex formations comprising high endothelial venules, B cell follicles with follicular DCs, GCs, and occasionally, lymphatic vessels (19). ELS can drive various B and T responses; however, their functionality is context-dependent. In autoimmune conditions such as multiple sclerosis (MS) and rheumatoid arthritis, ELS is associated with disease exacerbation (22, 25, 26). In contrast, in many cancer types, ELS exerts a favorable impact on overall survival and disease-free survival of patients (27–29). Nevertheless, the exact role of ELSs is not fully understood in the context of aging and AD-related neurodegeneration.

Here, we characterize ELS in the meningeal dural sinuses of aging and transgenic mouse models representing different aspects of neurodegenerative disorders. These include models overexpressing mutated human Aβ (such as 5xFAD and APP/PS1), commonly used to study AD, and a mutated human Tau strain (K257T/P301S) related to frontotemporal dementia (FTD). A mouse strain modeling DS, Dp1Tyb, that carries a duplication on chromosome 16 that is orthologous to human chromosome 21 and includes the APP and Dyrk1a genes (30, 31), was also studied to assess the combined effect of amyloid and tau pathologies. We show an age-dependent increase in ELS numbers and sizes in males older than 18 mo, whereas females showed a peak at 12 mo. Human APP- and PS1-related pathology in APP/PS1 mice reduced ELS numbers. In contrast, human APP-related pathology in 5xFAD mice exhibited increased ELS numbers. In models with hyperphosphorylated human Tau pathology and APP-related pathology in DS, there was a marked decrease in ELS accumulation. Together, these findings support the growing evidence that the meningeal immune environment is important in AD-related pathology.

## Results

### ELS Formation in Meningeal Dural Sinuses.

**Fig. 1.**
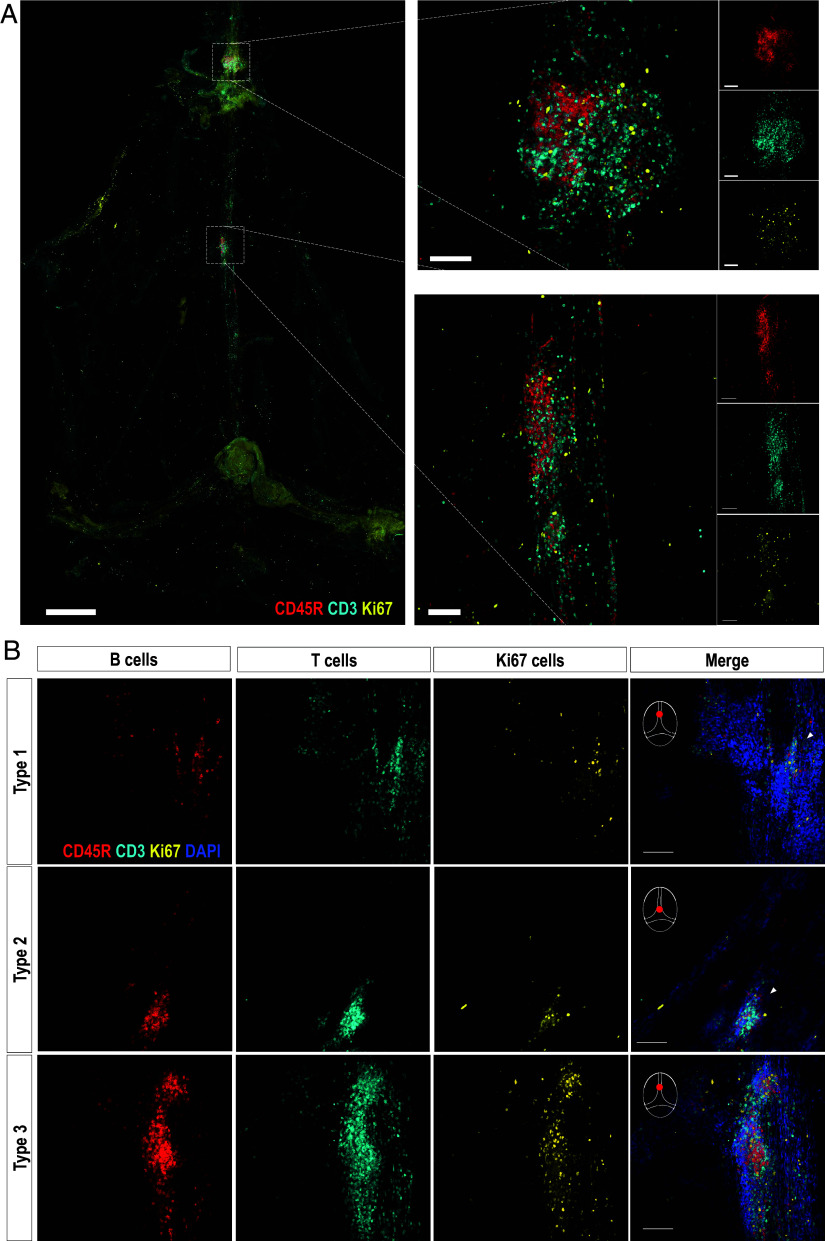
Meningeal ELS in aged mice. (*A*) Lymphatic structures in the Dural meninges of a 12-mo-old male APP/PS1 mouse, containing B cells (CD45R^+^ cells, red), T cells (CD3^+^ cells, cyan), and proliferating cells (Ki67^+^ cells, yellow). (Scale bar: *Left*, 2 mm; *Center* and *Right*, 100 µm.] (*B*) Lymphatic structures in the meninges, containing B cells (CD45R^+^ cells), T cells (CD3^+^ cells), and proliferating cells (Ki67^+^ cells), exhibit three phenotypes termed type 1 (top row), type 2 (middle row), and type 3 (bottom row).

A search for immune cell aggregations in the dura sinuses, which could indicate an active local immune response, revealed the presence of adaptive immune cells. Using immunofluorescence (IF) staining for B cells (CD45R^+^), T cells (CD3^+^), and the proliferation marker Ki67^+^, these cells form follicles along the sinuses. These follicles occasionally exhibit proliferation and different sizes and shapes. (Fig. 1*A*). This cellular composition suggests that these are meningeal ELS (18, 19, 27). Notably, the quantity and complexity of lymphatic structures are also important in assessing the stage and prognosis of various cancers, such as non–small cell lung cancer (32). In such cases, assessing the severity of the disease through lymphatic involvement is essential for effective clinical management (32–34). For this purpose, several scoring methods were developed to evaluate ELS predominantly in the context of tumor diagnosis (32, 35). Specifically, these methods assess ELS number, their complexity, the presence of GCs, and spatial localization within the tumor (32, 35, 36). Since the current study focuses on meningeal ELSs in the context of healthy aging rather than tumor-related ELS, we adapted these methods to generate a scoring system incorporating the quantity and complexity of the ELS in each mouse. To demonstrate their varying complexities, we classified ELS into three distinct phenotypes. Type 1 ELS can harbor either B cells, T cells, or both, arranged in a noncompartmentalized manner. Type 2 ELS comprises B and T cells in structurally compartmentalized regions, with little to no proliferation and lacking a defined shape. Type 3 ELS exhibits follicles expressing B and T cell markers, with some proliferating cells that express the proliferation marker Ki67^+^, organized into a B cell zone surrounded by T cells. These structures closely resemble GC structures (Fig. 1*B* and Movie S1).

### Aging Is Positively Correlated with Meningeal ELS Formation.

**Fig. 2.**
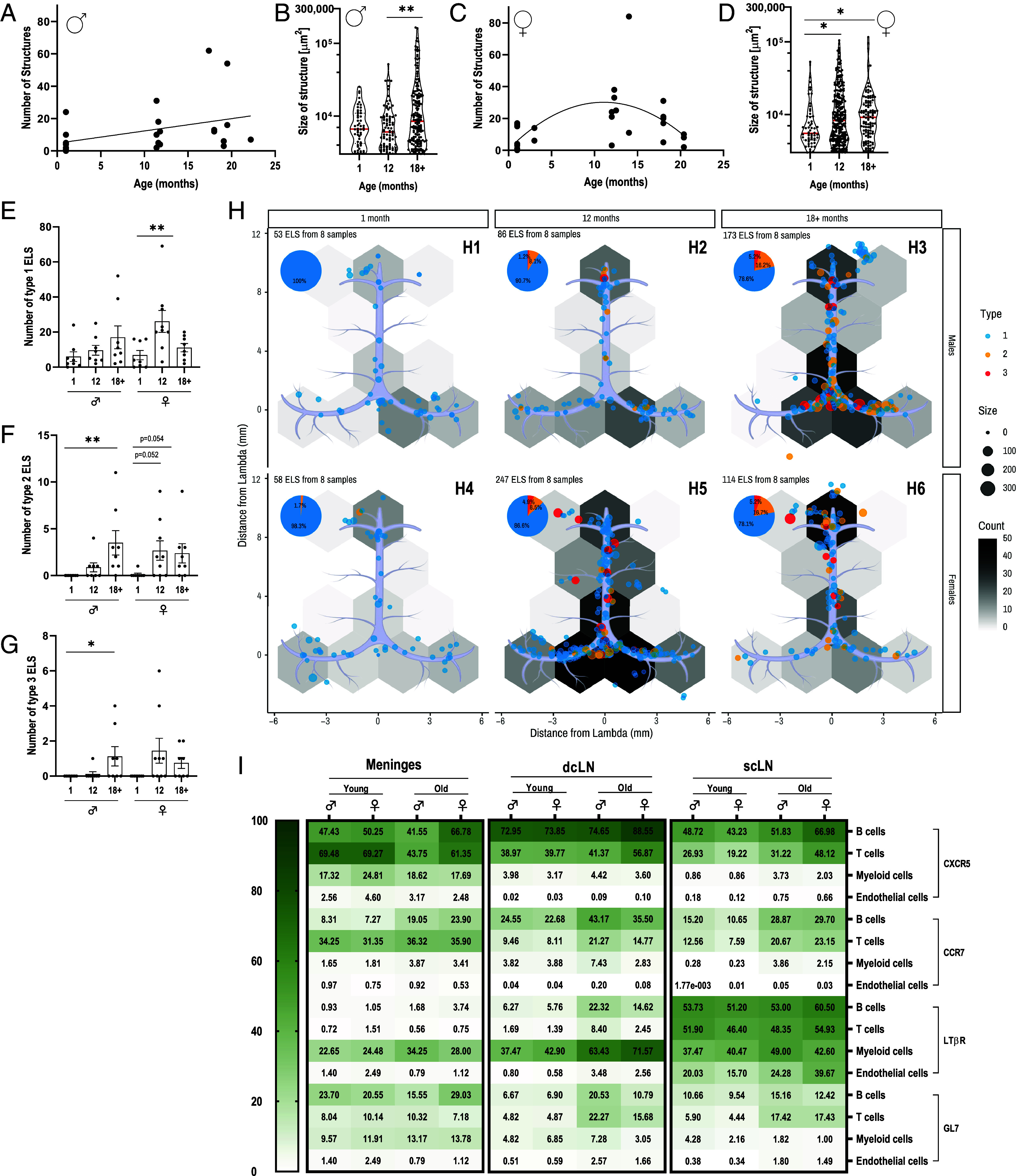
Formation of ELS correlates with age in C57bl/6 mice. Dural wholemounts from male and female C57bl/6 mice aged 1, 12, and >18 mo (n = 8 to 9 in each group) were analyzed for the presence of ELS. (*A*) Correlation between the number of ELS and age in naïve C57bl/6 males. Spearman’s r = 0.43, P < 0.05. (*B*) Violin plot of ELS size (μm^2^) of all individual structures in the three age groups of male mice. The median is represented as a red line. (*C*) No correlation was found between the number of ELS and age in naïve C57bl/6 female mice; hence, a nonlinear regression was computed. Goodness of fit R*^2^* = 0.33. (*D*) Violin plot of ELS size (μm^2^) of all individual structures in the three age groups of female mice. The median is represented as a red line. (*E*–*G*) Number of ELS of the three age groups in males and females for (*E*) type 1, (*F*) type 2, and (*G*) type 3. Kruskal–Wallis, *P < 0.05, **P < 0.01. (*H*) ELS Density map in the Dural sinuses of 1, 12, and >18-mo-old males (*H*_1_–*H*_3_) and females (*H*_4_–*H*_6_), respectively. The size of the circle represents the size of each structure, the density of the structures in each hexagonal area is represented by a gray scale between 0 and 50, and the phenotype of each structure is represented by blue for type 1, orange for type 2, and red for type 3. (*I*) Flow cytometric analysis of immune cell populations, expressing ELS-related markers (CXCR5, CCR7, LTβR, and GL7) in three different tissues: meninges, dcLN, and scLN. The analyzed population includes B cells (CD45R^+^), T cells (CD3^+^), myeloid cells (CD11b^+^), and endothelial cells (CD31^+^). The color legend indicates the percentage of cells positive for the ELS marker (depicted at far right) within the respective cell population. Sample size is n = 6 per group. Age groups include: Young = 1 to 3 mo and Old = 18+ mo.

To date, meningeal ELS has been described primarily in the context of brain pathologies such as MS (37–39). As dural ELS were recently shown to increase in aging (18), we sought to investigate the kinetics of this phenomenon and whether gender affects ELS formation. To this end, we analyzed dural wholemount tissues from male and female wild-type (WT) mice aged 1, 12, and >18 mo using IF staining for B cells (CD45R^+^), T cells (CD3^+^), and the Ki67^+^. Our analysis indicates that WT male mice exhibit an age-dependent increase in the number of meningeal ELS from 6 ± 2.79 at 1 mo (n = 8) to 10.75 ± 3.44 at 12 mo (n = 8) and 21.63±8.11 at >18 mo (n = 8) (Fig. 2*A* and *SI Appendix*, Fig. S1*A*). Total ELS area per meninges similarly exhibited an age-dependent increase between age groups (*SI Appendix*, Fig. S1*B*). The individual size of these structures increases correspondingly with age, although most structures are relatively small (Fig. 2*B*). Female mice, however, exhibit a bell-shaped age-dependent change in meningeal ELS numbers (Fig. 2*C*). Specifically, ELS numbers increase from 7 ± 2.68 at 1 mo (n = 8) to 30.22 ± 7.66 at 12 mo (n = 9) followed by a decrease to 14.25 ± 3.43 at >18 mo (n = 8) (Fig. 2*C* and *SI Appendix*, Fig. S1*A*). A similar effect is observed for the total ELS area (*SI Appendix*, Fig. S1*C*). The size distribution of these structures revealed that single ELS sizes increase between age groups (Fig. 2*D*). At 12 mo, females show more ELS than males (30.22 ± 7.66 and 10.75 ± 3.44, respectively, *SI Appendix*, Fig. S1*D*) and have larger ELSs compared with males (*SI Appendix*, Fig. S1*E*).

Regarding ELS complexity during aging, males develop increasingly complex structures, with a greater prevalence of types 1, 2, and 3 ELS, all showing similar trends of increased complexity over time (Fig. 2 *E*–*G*). In contrast, female mice exhibit a significant increase in type 1 ELS at 12 mo (Fig. 2*E*), whereas type 2 ELS shows a trend to increase in older age groups (Fig. 2*F*), and type 3 did not increase significantly with age (Fig. 2*G*).

ELS density analysis validated that in males, the density, numbers, and total size of dural ELS increase with age (Fig. 2 *H*_1–3_). However, in females, the most significant increase in ELS density is observed at 12 mo and, to a lesser extent, at >18 mo (Fig. 2 *H*_4–6_). These structures were predominantly localized to the confluence of sinuses in 12-mo-old female mice (Fig. 2 *H*_5_), similar to males >18 mo (Fig. 2 *H*_3_). Additionally, there was an increasing density of structures in the rostral-rhinal sinus (at the edge of the sagittal sinus) with age (Fig. 2*H*).

Next, we explored meningeal ELS development and how this immune compartment differs from the associated draining deep cervical lymph nodes (dcLN) and superficial cervical lymph nodes (scLN). To this end, spectral fluorescent flow cytometry (FC) was performed on cells isolated from the meninges, dcLN, and scLN of young (1 to 3 mo) and old (>18 mo) male and female WT mice.

Chemokine (C-X-C) receptor 5 (CXCR5) and C-C motif chemokine receptor 7 (CCR7), both known to be involved in lymphocyte trafficking (40), were predominantly expressed by both CD45^+^CD45R^+^ B cells and CD45^+^CD3^+^ T cells in the meninges, dcLN, and scLN (Fig. 2*I* and *SI Appendix*, Fig. S2 *A*–*C*). Notably, CCR7 expression increased in an age-dependent manner (Fig. 2*I* and *SI Appendix*, Fig. S2 *D*–*F*).

Lymphotoxin-β (LTβ) receptor (LTβR), which initiates ELS formation in inflamed tissues (41), was expressed by CD45^+^CD11b^+^ myeloid cells in all three immune tissues, suggesting a potential role for these cells in meningeal ELS formation. By contrast, CD31^+^ endothelial cells exhibit little to no LTβR expression (Fig. 2*I* and *SI Appendix*, Fig. S2 *G*–*I*). Importantly, and consistent with our observation of an age-dependent increase in meningeal ELS formation, both male and female WT mice showed an age-dependent upregulation of LTβR expression in CD11b^+^ cells, suggesting an age-dependent increase in meningeal lymphoid activity (Fig. 2*I* and *SI Appendix*, Fig. S2*G*). In addition, LTβR^+^ expression was observed in CD11b+ cells from the dcLN but not from the scLN, suggesting an age-dependent role in the draining dCLN (Fig. 2*I* and *SI Appendix*, Fig. S2 *H*–*I*).

The GL7 epitope is an α2,6‐linked sialic acid glycan that is exposed on the surface of activated B cells and T follicular helper cells in GCs, where it is involved in B cell receptor (BCR) signaling. GL7 expression was differentially manifested in the three immune compartments. In the meninges, B cells were the predominant GL7-expressing cell type, with higher expression observer in females compared to males (Fig. 2*I* and *SI Appendix*, Fig. S2*J*). In the draining dCLN of old, but not young mice, both B and T cells expressed higher GL7 levels than myeloid and endothelial cells (Fig. 2*I* and *SI Appendix*, Fig. S2*K*). In contrast, in the scLN, T cells were the predominant GL7-expressing cell type in both old male and female mice (Fig. 2*I* and *SI Appendix*, Fig. S2*L*).

### Extensive Age-Dependent ELS Accumulation in 5xFAD Mice.

**Fig. 3.**
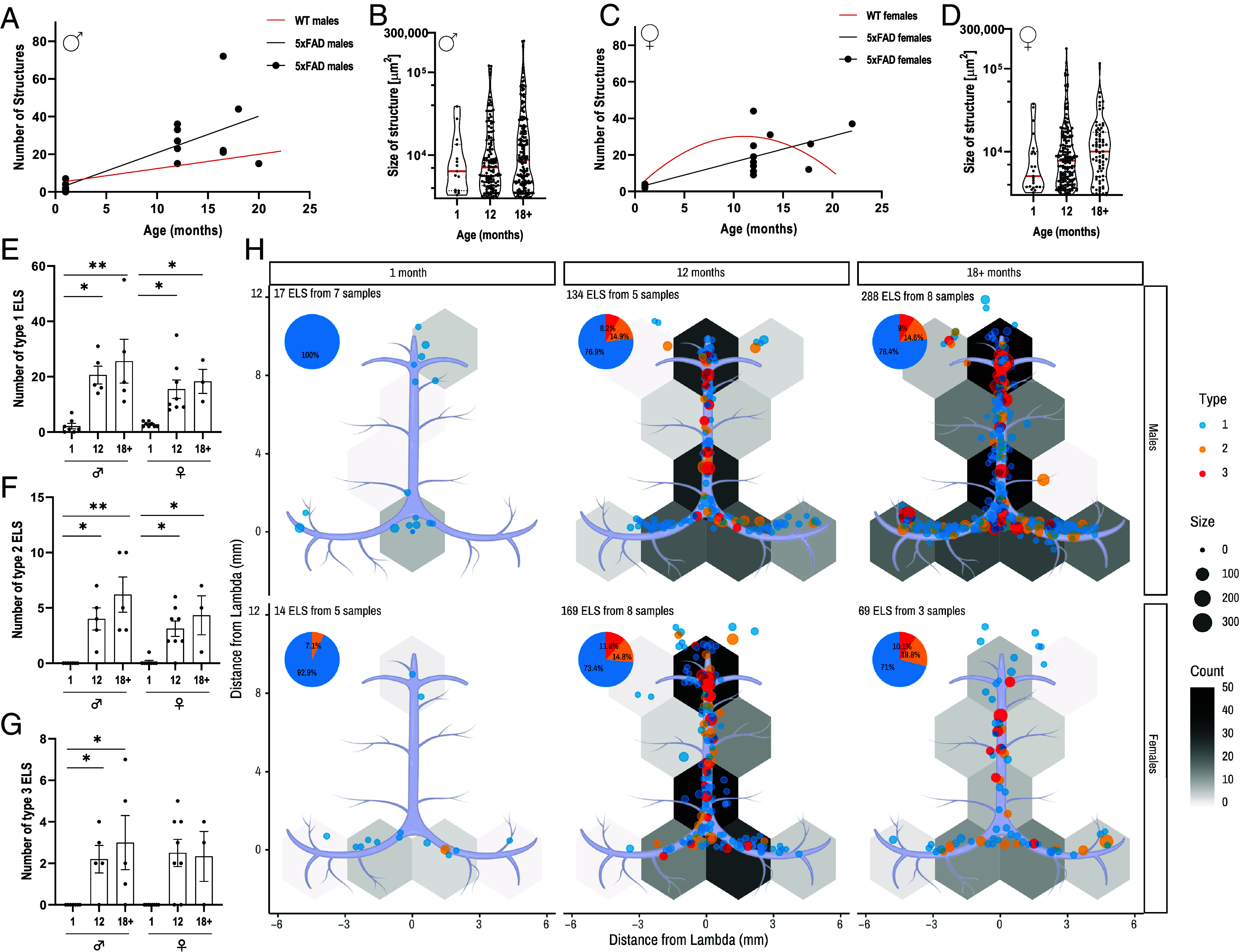
ELSs accumulate in higher quantities in an AD transgenic model. Dural wholemounts from male and female (n = 3 to 8 in each group) 5xFAD mice aged 1, 12, and >18 mo were analyzed for the presence of ELS. (*A*) Correlation between ELS numbers and age in 5xFAD males. The WT regression line is shown in red. Spearman’s r = 0.75, P < 0.001. (*B*) Violin plot of ELS size (μm^2^) of all individual structures in the three age groups of male mice. The median is represented as a red line. (*C*) Correlation between ELS numbers and age in 5xFAD females. The WT regression line is shown in red. Spearman’s r = 0.87, P < 0.0001. (*D*) Violin plot of ELS size (μm^2^) of all individual structures in the three age groups of female mice. The median is represented as a red line. (*E*–*G*) Number of ELS of the three age groups in males and females for (*E*) type 1, (*F*) type 2, and (*G*) type 3. (*H*) ELS Density map in the Dural sinuses of 1, 12, and >18-mo-old male (*H*_1_–*H*_3_) and female mice (*H*_4_–*H*_6_). Red lines in (*A*) and (*C*) indicate the C57bl/6 mice trendlines for reference. Kruskal–Wallis, *P < 0.05, **P < 0.01.

Following our observation of an age-associated increase in ELS formation in WT males and bell-shaped curve dynamics in WT females, we were interested in assessing whether 5xFAD mice, which manifest a relatively rapid age-dependent amyloid brain pathology and parenchymal inflammation, would exhibit differential regulation of these structures. Similar to WT mice, 5xFAD males show an age-associated increase in ELS formation. However, these structures accumulated more rapidly than in WT, from 2.14 ± 0.96 to 26.80 ± 3.72 and 34.80 ± 10.52 at 1 (n = 7), 12 (n = 5), and >18 mo (n = 5), respectively (Fig. 3*A* and *SI Appendix*, Fig. S3*A*). In addition, the total ELS area and structure sizes per meninges also increase with age compared to WT (Fig. 3*B* and *SI Appendix*, Fig. S3*B*). Unlike WT female mice that exhibited bell-curve dynamics, 5xFAD females exhibit a consistent age-dependent increase in dural ELS formation and size (2.87 ± 0.35 structures at 1-mo-old n = 8, 21.13 ± 4.15 structures at 12-mo n = 8, and 25 ± 7.23 structures at >18 mo n = 3, Fig. 3*C* and *SI Appendix*, Fig. S3 *A*–*C*). Older 5xFAD females also exhibit an age-dependent trend to an increase in individual structure size (Fig. 3*D*). Both 5xFAD males and females show an increased number of type 2 ELS in the older age groups, with males additionally showing a significant increase in type 3 ELS (Fig. 3 *E*–*G*). These structures are prominently located at the rostral-rhinal area and close to the confluence of sinuses (Fig. 3*H*), similar to the WT groups (Fig. 2*H*).

### ELS Formation Does Not Increase with Age in APP/PS1 Mice.

**Fig. 4.**
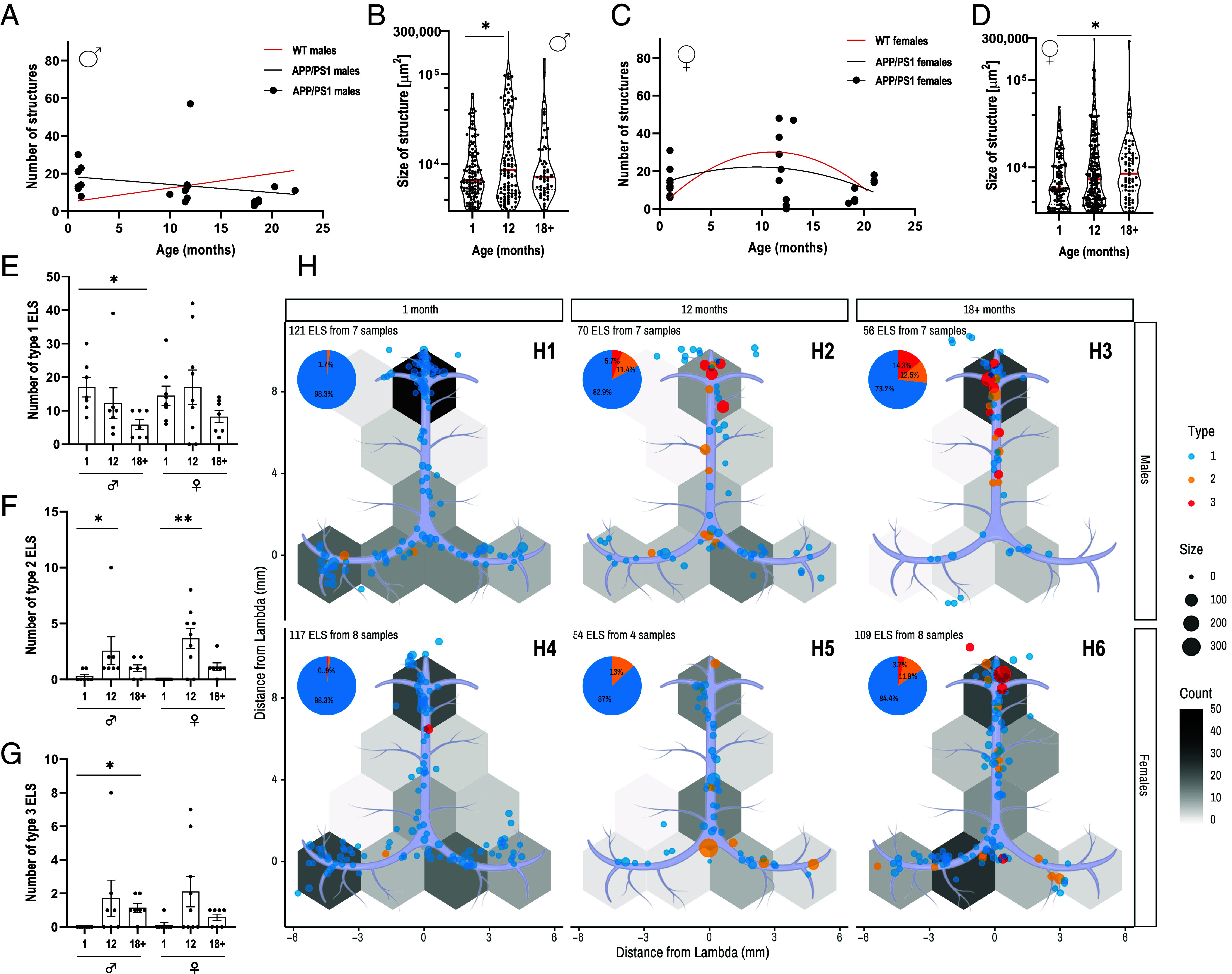
Formation of ELS correlates with age in APP/PS1 mice. Dural wholemounts from male and female (n = 7 to 9 in each group) APP/PS1 mice aged 1, 12, and >18 mo were analyzed for the presence of ELS. (*A*) Correlation between ELS numbers and age in APP/PS1 males. The WT regression line is shown in red. Spearman’s r = −0.38, P = ns. (*B*) Violin plot of ELS size (μm^2^) of all individual structures in the three age groups of male mice. The median is represented as a red line. (*C*) Nonlinear regression of ELS numbers and age in APP/PS1 females. The WT regression line is shown in red. Goodness of fit R squared = 0.11. (*D*) Violin plot of ELS size (μm^2^) of all individual structures in the three age groups of female mice. The median is represented as a red line. (*E*–*G*) Number of ELS of the three age groups in males and females for (*E*) type 1, (*F*) type 2, and (*G*) type 3. (*H*) ELS Density map in the Dural sinuses of 1, 12, and >18-mo-old male (*H*_1_–*H*_3_) and female mice (*H*_4_–*H*_6_). Red lines in (*A*) and (*C*) indicate the C57bl/6 mice trendlines for reference. Kruskal–Wallis, *P < 0.05, **P < 0.01.

To assess whether the effects observed in 5xFAD mice are related to amyloid pathology or strain-specific, we also evaluate APP/PS1 mice. Surprisingly, we observed an opposite effect when assessing ELSs in APP/PS1 mice, which also exhibit early-onset Aβ pathology. Our analysis indicates that in male APP/PS1 mice, an age-dependent decrease occurs in the number of dural ELS, from 17.29 ± 2.89 at 1 mo (n = 7) to 16.57 ± 6.84 at 12 mo (n = 7) and 8 ± 1.59 at >18 mo (n = 7) (Fig. 4*A* and *SI Appendix*, Fig. S4*A*). Young APP/PS1 males show significantly more structures in the dura than young WT (mean of 17.29 ± 2.89 compared to 6 ± 2.79, *SI Appendix*, Fig. S4*B*). The total ELS area exhibited no age-dependent increase, while WT mice showed a mild increase with age (*SI Appendix*, Fig. S4*C*). Further, the median ELS size increases significantly between 1 and 12 but not >18 mo old male APP/PS1 mice (Fig. 4*B*). Total ELS area exhibited no age-dependent increase, while WT mice showed a mild increase with age (*SI Appendix*, Fig. S4*C*). Further, the median ELS size in males increased significantly between 1 and 12 but not >18-mo-old male APP/PS1 mice (Fig. 4*B*). APP/PS1 Females, in contrast, exhibit an ELS formation pattern similar to WT (Fig. 4*C* and *SI Appendix*, Fig. S4*D*) (n = 8, 9, and 7 for 1, 12, and >18 mo, respectively). Further, female individual structure size increases between young and old age groups (Fig. 4*D*). With respect to ELS complexity, male APP/PS1 mice exhibit fewer type 1 structures but more type 2 and 3 structures during aging (Fig. 4 *E–G*). In contrast, females exhibit an increase in type 2 ELS between young and old mice but no significant changes in types 1 and 3 structures (Fig. 4 *E*–*G*). Type 1 ELSs are spread throughout the dural sinuses in males and females, whereas most complex structures accumulate at the rhinal dural sinus (Fig. 4*H*).

### Parenchymal Aβ Alone Is Not Responsible for Meningeal ELS Formation.

**Fig. 5.**
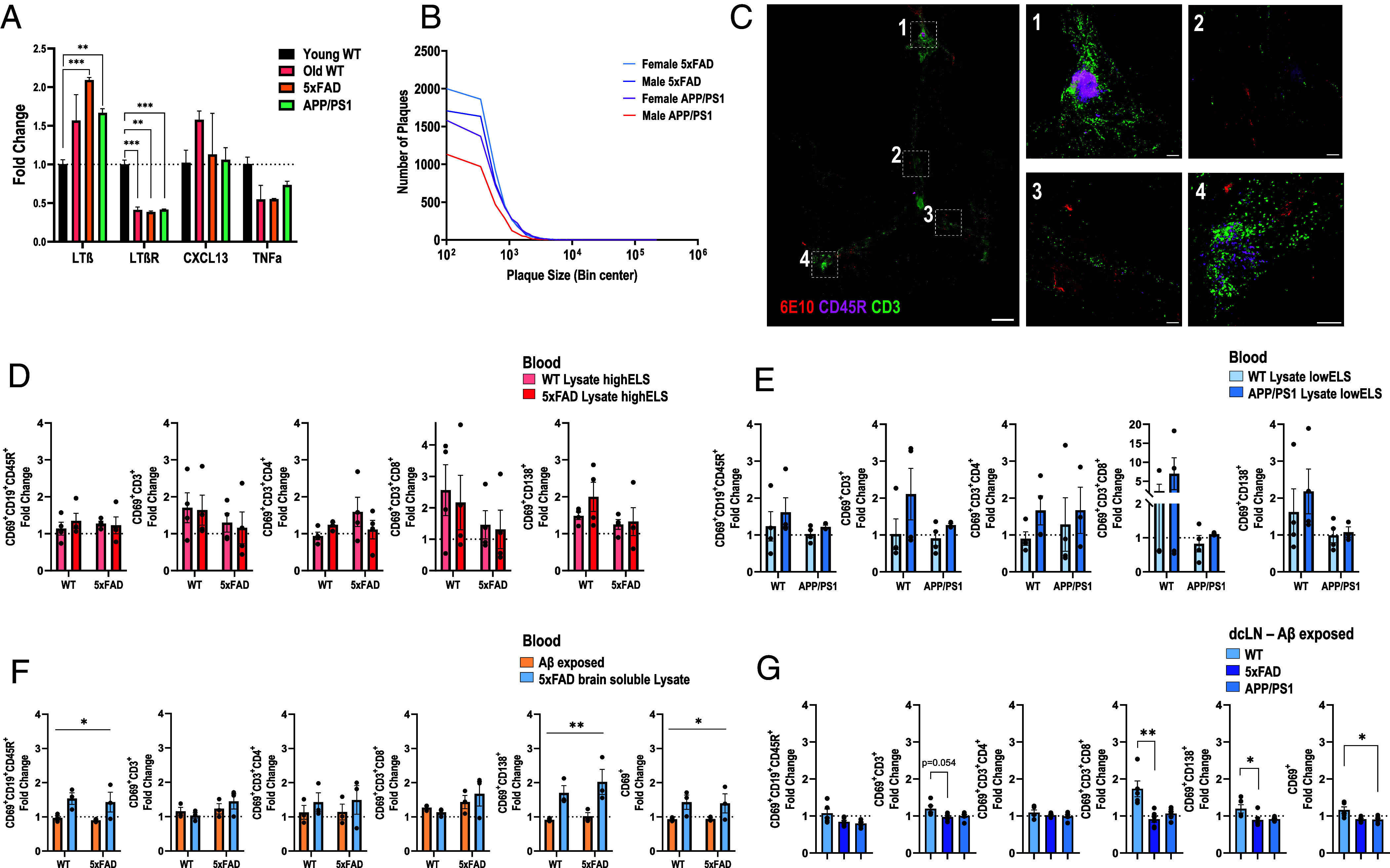
Relationship between Aβ pathology and the immune system in 5xFAD and APP/PS1 mice. (*A*) qPCR analysis of meninges from young (1 mo) WT, old (18 mo) WT, old 5xFAD, and old APP/PS1 male mice, assessing the expression of LTβ, LTβR, CXCL13, and TNFα. Gene expression is shown as fold change relative to the young WT group, n = 3, multiple t tests, **P < 0.01, ***P < 0.001. (*B*) Frequency distribution of individual plaque size (µm^2^) in 5xFAD and APP/PS1 males and females at 12 mo old. N = 4 per group. (*C*) Staining of plaques (red), CD45R^+^ B cells (pink), and CD3^+^ T cells (green) in a 20-mo-old 5xFAD male. (Scale bar, large image, 1 mm; small image, 100 µm). (*D*–*G*) FC analysis of activated (CD69^+^) PBMCs, including CD19^+^CD45R^+^ B cells, CD3^+^ T cells, CD3^+^CD4^+^ T helper cells, CD3^+^CD8^+^ cytotoxic T cells, and CD138^+^ plasma cells, cultured with either: (*D*) 5xFAD or WT soluble brain lysate with high ELS count in WT and 5xFAD 15- to 19-mo-old males. Kruskal–Wallis. All ns; (*E*) APP/PS1 or WT soluble brain lysate with low ELS count in WT and 5xFAD 15- to 19-mo-old males. Kruskal–Wallis.All ns; (*F*) FC analysis of 12-mo-old 5xFAD and WT male PBMC activated immune cells, expressed as fold change in response to culture with Aβ_42_ and 5xFAD brain soluble lysate. 2-way ANOVA, treatment effects; (*G*) FC analysis of dcLN cells of 17- to 19-mo-old M and F WT, 5xFAD, or APP/PS1 cultured with oligomeric Aβ_42_. Kruskal–Wallis. *P < 0.05, **P < 0.01.

To investigate whether aging and AD-related pathology influence the expression of genes associated with meningeal ELS formation, we analyzed the meninges of aged (>18 mo) and young (<2 mo) WT, 5xFAD, and APP/PS1 male mice. Our findings demonstrated that aged mice across all strains exhibited elevated transcription of LTβ with a concomitant decreased transcription of its receptor, LTβR, compared to young WT mice (Fig. 5*A*). In contrast, the transcriptional regulation of chemokine (C-X-C motif) ligand 13 (CXCL13) and tumor necrosis factor-α (TNF-α), two additional factors involved in ELS formation, did not differ significantly from those in young WT mice (Fig. 5*A*). These results support our hypothesis that aging, regardless of AD pathology, is associated with increased expression of certain ELS-related genes in the meninges. This transcriptional profile suggests that meningeal ELS formation may be driven by aging alone, even in the absence of amyloid pathology. To further delineate the relationship between ELS formation and Aβ pathology, we analyzed the plaque burden of both 5xFAD and APP/PS1 strains at 12 mo of age. To this end, coronal hippocampal slices from 5xFAD and APP/PS1 mice were immunostained for Aβ plaques (*SI Appendix*, Fig. S5 *A* and *B*). 5xFAD female mice exhibited a higher plaque burden compared with male 5xFAD. In contrast, female APP/PS1 mice exhibited higher plaque burden, compared with male APP/PS1 mice (Fig. 5*B*). Further, we aimed to determine whether Aβ aggregations dictate ELS formation in the meningeal dura. To this end, we visualized 6E10-positive meningeal Aβ aggregates, along with B and T cells, in a 20-mo-old 5xFAD male. Surprisingly, dural ELSs did not primarily form around plaques, and interactions between B and T cells were rarely observed despite the presence of large type 3 ELS in the rostro-rhinal region (Fig. 5*C*).

To investigate whether oligomeric Aβ or other parenchymal factors activate peripheral immune cells, leading to ELS formation, we isolated lymphocytes from blood and draining dcLN from aged WT, 5xFAD, and APP/PS1 mice (*SI Appendix*, Fig. S5*C*). Cells were stimulated with either Phorbol 12-myristate 13-acetate (PMA), as a positive control (*SI Appendix*, Fig. S5*D*), oligomeric recombinant human Aβ_42_, or brain lysates obtained from WT, 5xFAD, or APP/PS1 mice, obtained from meningeal tissues with either low or high ELS numbers. Brain lysates derived from 5xFAD and APP/PS1 mice were used to model exposure of circulating immune cells to a complex mixture of oligomeric, fibrillar, and plaque forms of amyloids that may transfer from the parenchyma to the meninges. Lysates derived from WT mice served as a baseline control.

Peripheral blood mononuclear cells (PBMCs) from 5xFAD mice, incubated with brain lysates of 5xFAD or WT brains, were not differentially activated compared to PBMCs from WT mice (Fig. 5*D*). Similarly, PBMCs of APP/PS1 mice incubated with soluble brain lysates from either APP/PS1 or WT mice were not differentially activated compared to PBMCs from WT mice (Fig. 5*E*). These findings suggest that parenchymal factors alone are insufficient to include differential activation of circulating lymphocytes in aged mice and thus cannot plausibly explain the discrepancy in ELS formation observed between the strains. We next examined whether Aβ_42_ differentially activates PBMCs compared to brain lysate. Overall, 5xFAD-derived lysate induced a more effective immune activation in both WT- and 5xFAD-derived PBMCs than oligomeric human Aβ_42_, as indicated by the proportion of activated (CD69^+^) cells (Fig. 5*F*), indicating that Aβ_42_ within the lysate is not the primary driver of PBMC stimulation by brain lysates. To determine whether oligomeric Aβ_42_-mediated immune cell activation could occur in a more localized compartment, we stimulated immune cells derived from the draining dcLN. Surprisingly, Aβ_42_ induced stronger activation in WT lymphocytes compared to those from 5xFAD and APP/PS1 mice (Fig. 5*G*). Together, these findings suggest that in EOAD mice, meningeal lymphocytes are not directly reactive to Aβ_42_. Instead, their recruitment to the meningeal compartment is likely driven by proinflammatory factors that trigger immune activation, promoting ELS formation.

### The K257T/P301S Tau Mutation Confers Reduced Meningeal ELS Formation.

In contrast to mouse models of EOAD, mice carrying the K257T/P301S double mutation in the human Tau gene (associated with frontotemporal dementia and tauopathy but lacking the expression of human Aβ plaques) revealed remarkably low numbers of meningeal ELS in >18-mo-old females compared with WT mice (5.40 ± 2.18 and 14.25 ± 3.43, respectively; *SI Appendix*, Fig. S6*A*). Accordingly, the total area of the structures was significantly smaller in the mutated mice compared with WT mice (mean of 5.3 × 10^3^ n = 10 and 18 × 10^4^ n = 8, respectively. *SI Appendix*, Fig. S6*B*). The number of type 1, 2, and 3 structures were significantly lower compared with WT mice (*SI Appendix*, Fig. S6 *C*–*E*). The unexpectedly low presence of ELS in old K257T/P301S mice (*SI Appendix*, Fig. S6*F*) suggests an inverse association between meningeal ELS and Tau pathology.

### Meningeal ELS Formation Is Reduced in Dp1Tyb Mice.

Having observed a reduction in ELS numbers and sizes in mice carrying the K257T/P301S tau mutation, we next assessed whether ELS formation is also altered in Dp1Tyb mice, which model DS (31). This is particularly relevant, as individuals with DS typically exhibit both amyloid and tau pathology (42, 43). Surprisingly, Dp1Tyb mice aged 10 to 15 mo exhibited significantly fewer ELS than 12-mo-old WT mice. In males (n = 13), ELS numbers were 4.53 ± 1.09 in Dp1Tyb mice vs. 10.75 ± 3.44 in WT mice (*SI Appendix*, Fig. S7*A*). In females (n = 12), we observed 2.25 ± 0.66 ELS in Dp1Tyb mice and 30.22 ± 7.66 in WT mice (*SI Appendix*, Fig. S7 *B* and *C*). The total ELS area and the median size of individual ELSs did not differ in Dp1Tyb compared to WT males (*SI Appendix*, Fig. S7 *D* and *E*). However, females showed significantly less total ELS area than WT (*SI Appendix*, Fig. S7 *F* and *G*). Concerning ELS type, 10- to 15-mo-old male and female Dp1Tyb mice exhibited more type 1 structures than types 2 and 3 (*SI Appendix*, Fig. S7 *H* and *I*). Notably, female Dp1Tyb mice had no type 3 ELS (*SI Appendix*, Fig. S7*I*). ELS density analysis confirmed that overall, dural ELS decreased in females but not males in Dp1Tyb mice compared to WT mice (*SI Appendix*, Fig. S7*J*). The Dp1Tyb strain exhibits elevated Aβ protein production (up to 1.3-fold) but does not form plaques (31). To analyze parenchymal pathology, we measured the concentration of the cortical and hippocampal mouse Aβ_42_ and Aβ_40_ peptides in their soluble and insoluble fractions. The correlation between ELS and Aβ-related pathology in Dp1Tyb mice revealed that the total ELS area is positively correlated to insoluble mouse Aβ_42_ levels in the hippocampus of Dp1Tyb males (Spearman’s *r* = 0.89, *P* = 0.014, *SI Appendix*, Fig. S7*K*). Thus, although DP1Tyb mice exhibit elevated Aβ_42_ levels, ELS formation remained blunted, possibly due to the widespread immune dysregulation characteristic of DS individuals, as well as in DP1Tyb mice.

## Discussion

The meningeal compartment plays a key role in both brain homeostasis and pathology (9, 31, 32). The present study provides evidence that dural ELS, predominantly located near the sinuses, are differentially regulated by age, sex, and CNS pathology. These findings emphasize the dynamic interactions between the meningeal compartment, the surrounding periphery, and, critically, the brain itself.

The physiology and integrity of the meninges change in both humans and rodents with aging (9, 12, 44–47). Here, we report that adaptive immune cells from ELS are present in greater numbers in aged, otherwise healthy, WT mice. The mechanism of this phenomenon may be driven by age-related immune dysregulation, including the secretion of chemokines and cell-activating cytokines linked to inflammaging, or age-associated meningeal B cell accumulation. Aging is also associated with extracellular matrix remodeling, which impairs cerebrospinal fluid (CSF) drainage and slows blood flow through the dural sinuses (47), while simultaneously increasing the vasculature area, vessel, and sinus diameter (48). Additionally, synchronized neuronal activity during sleep was shown to promote CSF flow via the glymphatic system (49, 50), suggesting that age-associated sleep impairment would limit neuronal synchronization, promote the accumulation of pathological factors in the brain, resulting in inflammaging. Together, these mechanisms may underlie the age-associated increase in ELS formation.

Previous studies have shown that sinus-associated meningeal ELSs can emerge following transient microbial infections, including bacterial, viral, and fungal exposures (18). Our study extends these findings by demonstrating that lymphocyte activation can also occur following exposure to parenchymal factors under sterile inflammatory conditions. Importantly, activation of peripheral immune cells was not driven by brain-derived Aβ, showcasing the role of the broader parenchymal environment in shaping meningeal immunity. This aligns with previous findings linking specific immune subsets and parenchymal cells (13, 51, 52). However, the mechanisms by which aging and Aβ-related pathology influence immunoglobulin class switching remain unknown and warrant future investigation to clarify the role of ELS in neurodegeneration.

In our study, we also investigated molecular changes related to ELS formation. Alterations in cytokine expression and immune trafficking pathways, including CCR7, CXCR5, LTβ, and LTβR, were observed in aging and AD models. Targeting these pathways could offer tools to modulate meningeal ELS formation and investigate their functional impact on the CNS (53, 54).

As mentioned above, and recently reviewed (21), meningeal ELS have been primarily described in the context of brain pathologies such as MS (37–39, 55), and both meningeal and parenchymal ELS have been reported in gliomas (21, 56–58). Comparing our observations with these reports reveals both shared and distinct features of ELS across different pathological contexts (*SI Appendix*, Table S1). In MS, ELS formation can be experimentally induced by self-antigen immunization, and it functions as a center of B cell expansion and plasma cell development against brain antigens. This process results in a higher number of parenchymal lesions and demyelinated regions in both humans and mice (21, 55). In contrast, ELS in glioma are associated with T cell infiltration into the tumors, although their association with favorable disease outcomes, compared to other malignancies, remains inconclusive (21, 57). These examples showcase how the pathological context underlies ELS function, emphasizing the importance of understanding their role in AD.

This complexity is further illustrated by the contrasting patterns of ELS formation observed in the 5xFAD and APP/PS1 strains, which is not fully understood, despite their high similarity in expressed mutations and amyloid pathology. In previous studies, the 5xFAD (strain #34840, Jackson Laboratories) and PSAPP (strain #034829, Jackson Laboratories) strains, both carry the HuAPP695swe APP mutation, albeit differ in their human PS1 mutation (M146L and L286V in 5xFAD and ΔE9 in PSAPP), exhibited contrasting outcomes following RAG2 deficiency-mediated adaptive immune depletion. Specifically, B and T cell depletion led to increased parenchymal Aβ loads in 5xFAD mice (16), whereas in PSAPP (17) and APP/PS1 (15) mice (Swedish mutation APPKM670/671NL and the presenilin 1 mutation, PS1I166P), Aβ burden was reduced. These findings indicate that differences in adaptive immunity between these strains can either suppress or aggravate AD pathology, depending on the strain, possibly due to differences in microglial phagocytosis response. Specifically, in 5xFAD mice, B cells secrete higher levels of preimmune IgGs than WT, resulting in elevated microglial phagocytosis of plaques, and depletion of adaptive immune cells in these mice blunts microglial Aβ phagocytosis. In contrast, depleting adaptive immune cells in PSAPP mice resulted in elevated microglial-mediated Aβ phagocytosis.

PS1 plays a role in regulating microglial activity, and different PS1 mutations can lead to distinct effects on microglial function (59–62). The discrepancy observed between 5xFAD and APP/PS1 in our study could be attributed to the differential effects these mutations potentially exert on microglial functionality.

Our data suggest that the increased presence of dural ELS in 5xFAD mice is unlikely to result from an enhanced capacity of peripheral immune cells to respond to Aβ or other stimuli. Instead, chronic drainage of neurodegenerative proteins from the 5xFAD brain to meningeal and peripheral lymphoid tissues may drive ELS accumulation. It remains unclear whether parenchymal proinflammatory factors directly activate meningeal stromal cells to initiate ELS formation, or whether dcLN lymphocytes are recruited to the meninges, where they populate it and trigger inflammation leading to ELS formation. In our work, Aβ_42_-mediated immune cell activation in the dcLN-derived cells suggests that WT mice may retain a more responsive local immune milieu in the dcLN, whereas chronic Aβ exposure in 5xFAD and APP/PS1 mice could lead to immune tolerance.

Interestingly, unlike the 5xFAD and APP/PS1 strains, which express mutated human Aβ, mice expressing the FTD-related K257T/P301S tau mutation and Dp1Tyb mice modeling DS, exhibit fewer ELS than age-matched WT mice. In the case of Dp1Tyb mice, this may be attributed to the widespread immune dysregulation associated with individuals with DS (63) and in corresponding mouse models (31). This dysregulation includes innate immune cells, adaptive immune B and T cells, and immunoglobulin class switching and expression, ultimately leading to an impaired immune response to various pathologies. Such impairments have been observed in both human and mouse models of DS (31, 63, 64), including the Dp1Tyb strain (65).

More enigmatic is the reduced ELS formation in the K257T/P301S tau mice. Notably, levels of proinflammatory cytokines including IL-6, IL-1β, and TNF-α, are elevated in K257T/P301S tau mice compared to WT controls (66, 67). Thus, despite a low-grade neuroinflammatory environment typical of tauopathy, ELS numbers remained low, and the underlying cause remains to be studied.

Sex differences in immune function also appear to influence ELS development. Although there is no significant difference in blood flow rate in the sinuses of WT males and females (68), females demonstrate greater B cell activation and antibody responses (65). Additionally, females have higher numbers of GC B cells containing superior somatic hypermutation frequencies compared to males (69). These differences are largely regulated by sex steroids, such as estrogens (65). Understanding the causes for this discrepancy could provide insights into why women are nearly twice as likely to develop AD compared to men (70).

In support of this, aging in 5xFAD mice exhibits sex-specific interactions between hormonal changes and AD pathology, though strain-specific hormonal differences remain undercharacterized. Female 5xFAD mice demonstrate heightened AD-like pathology compared to males, including increased cortical Aβ plaque burden between 4 and 18 mo of age (71, 72), elevated neuroinflammation (IL-1β, TNFα, astrocyte reactivity) (73, 74), and exacerbated Aβ accumulation after perimenopause (72). While loss of ovarian hormones in midlife female mice correlates with hippocampal synaptic loss and cognitive decline (75), direct measurements of systemic estradiol/progesterone in aged 5xFAD vs. WT mice are lacking. Paradoxically, male 5xFAD mice show elevated testicular testosterone versus WT mice (71), suggesting complex strain–sex interactions. In humans, rising follicle-stimulating hormone (FSH) levels during menopause are associated with increased Aβ deposition in the frontal cortex and gray matter loss (76), implicating hypothalamic–pituitary–gonadal axis dysregulation in AD risk. Although similar mechanisms are plausible in mice, gonadotropin levels in 5xFAD mice remain understudied. Critically, sex differences in neuroinflammation are well documented in AD. Women show stronger correlations between tau pathology and translocator protein (TSPO) density (a neuroinflammation marker) than men (74). In 5xFAD mice, this aligns with female-specific hyperactivity and deficits in social exploration (71), suggesting that hormonal-immune pathways crosstalk in these model mice.

The findings described herein provide insight into the complex role of the adaptive immune system in neurodegenerative diseases at the meningeal interface. Our study shows evidence that ELS immune hubs accumulate with age in the meninges of WT and 5xFAD mice, and this process is sex dependent. Additionally, the observation that not all CNS pathologies lead to a clear, unidirectional effect on ELS formation suggests a more complex interaction between brain pathology, CNS inflammation, and peripheral immune activation.

## Methods

Extended methods can be found in *SI Appendix*, *Methods*. Below is a brief description of the methods.

### Animals.

All mice were housed in an SPF facility at Bar-Ilan University under standard conditions. Animal care, genotyping, and experiments were approved by ethical committees. Wild-type C57bl/6 mice were used as controls, while the 5xFAD and APP/PS1 strains modeled EOAD, expressing mutations in APP and PS1. The Tau double mutant (K257T/P301S) was used to model Tau hyperphosphorylation, and the Dp1Tyb strain modeled APP-related pathology in DS.

### Immunofluorescence of Meningeal Wholemounts.

Mice were perfused with ice-cold PBS, and skulls were preserved with 2% PFA. After blocking, tissues were incubated with primary antibodies against CD3 and Ki67, followed by secondary antibody staining. Meningeal tissue was then stained with anti-CD45R and Hoechst before being mounted for imaging. Slides were scanned using The Opera Phenix® Plus High-Content Screening Confocal System by Revvity. Images were analyzed with FIJI software using a custom macro to classify ELS.

### Amyloid Plaque Analysis.

Hemispheres of 5xFAD and APP/PS1 brains were fixed, cryosectioned, and stained with anti-Aβ antibody (6E10). Sections were then stained with Hoechst and mounted on slides for imaging using The Opera Phenix®. Image analysis was performed using FIJI software to measure the size and quantity of plaques.

### Meningeal ELS Density Map.

A reproducible R pipeline was used to analyze the density of meningeal ELS. The data were processed using the R statistical language v4.4 (R Core Team, 2024). _R: A Language and Environment for Statistical Computing_. R Foundation for Statistical Computing, Vienna, Austria. Results were visualized with the ggplot2 and patchwork packages. The analytical scripts are available at https://zenodo.org/records/15453928 under an Artistic 2.0 license (77).

### FC.

The Meninges and cervical lymph nodes were collected and suspended following perfusion with ice-cold PBS. Cells were stained for CD45, CD45R, CD3, CD11b, CD31, CD185 (CXCR5), CD197 (CCR7), LTβR, GL7, and a viability marker.

### For Activation of PBMCs and LN.

Blood was collected from the facial vein, and PBMCs were isolated. Cervical lymph nodes were collected and suspended following perfusion. PBMCs were activated with either oligomerized human Aβ_42_ or brain lysates. Lymph nodes were activated with oligomerized human Aβ_42_. Cells were stained for CD3, CD4, CD8, CD19, CD45R, CD69, CD138, and a viability marker. Samples from both cohorts were recorded on a Cytek® Aurora spectral flow cytometer. An equal number of live cells was exported from each sample within tissues and analyzed in FlowJo software v10.10.0.

### Brain Lysate Preparation.

Mouse brains were homogenized and centrifuged to prepare tissue lysates. The total protein concentration was determined using a BCA assay, and lysates were normalized for use in activation assays.

### RT-PCR.

RNA was extracted from Meninges, cDNA was synthesized, and quantitative RT-PCR was performed to assess gene expression on a StepOnePlus Real-Time PCR Systems (Applied Biosystems™). Primers for actin, LTβ, LTβR, CXCL13, and TNFα were used for analysis. Fold change ratios were calculated using the Pfaffl method to quantify expression levels (78).

### Sandwich ELISA for Mouse Aβ Levels.

96-well plates were coated with anti-Aβ antibodies, blocked, and incubated with tissue lysates. After washing, detection antibodies were applied, followed by substrate addition to measure optical density. Levels of Aβ_40_ and Aβ_42_ were calculated as protein mass of total protein per sample (μg/mg).

### Statistics.

Power analysis was conducted using G*Power to assess study power. Data were analyzed using ANOVA, t-tests, and nonparametric tests as appropriate, with Holm–Sidak adjustment for multiple comparisons. Statistical tests were performed using GraphPad Prism version 8.4.2 for Windows, GraphPad Software, Boston, MA, www.graphpad.com. Statistical significance was set at *P < 0.05.

## Supplementary Material

Appendix 01 (PDF)

Movie S1. 3D video of a type 3 lymphoid structure in the sagittal sinus. CD45R+ B cells (red), CD3^+^ T cells (cyan), and Ki67^+^ cells (yellow).

## Data Availability

Software code have been deposited in Zenodo (https://zenodo.org/records/15453928) (77). All other data are included in the manuscript and/or supporting information.
