# Supplementary material for: Dural ectopic lymphatic structures accumulate during aging and exhibit dysregulation in neurodegenerative diseases
Source: Proc Natl Acad Sci U S A. 2025 Aug 12;122(33):e2425081122. doi: 10.1073/pnas.2425081122 (PMC12377736; doi:10.1073/pnas.2425081122)
Supplement: Supplementary file 1 — Appendix 01 (PDF) [file pnas.2425081122.sapp.pdf]

A

	WT					
	Males			Females		
	1 month	12 months	18+ months	1 month	12 months	18+ months
Number of mice	8	8	8	8	9	8
Minimum ELS number	0	2	3	0	3	2
Maximum ELS number	24	31	62	17	84	31
Mean number of ELS per group	6	10.75	21.63	7	30.22	14.25
Std. Dev	7.91	9.75	22.93	7.597	22.99	9.706
SEM	2.797	3.447	8.108	2.686	7.664	3.432

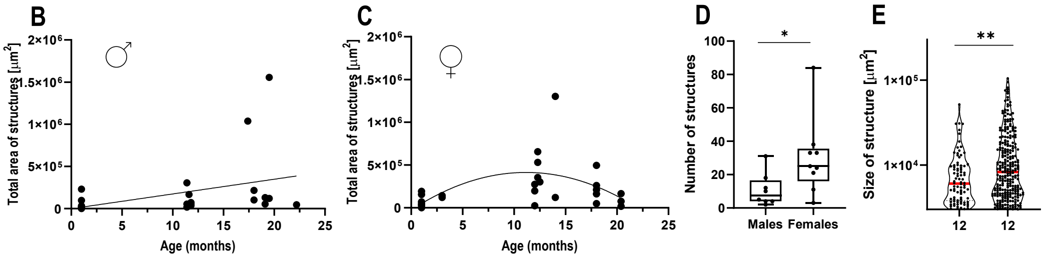

Figure S1. (**A**) Descriptive statistics for the number of ELS per group in the WT (C57bl/6) groups. (**B**) Correlation of total ELS area (mm^2) and age and linear regression in naïve C57bl/6 male mice. *Spearman's r=0.52, p<0.01*. (**C**) Non-linear fit regression of total ELS area (mm^2) and age in naïve C57bl/6 female mice. (**D**) 12-month-old WT females have more meningeal ELS than age-matched males. *Mann-Whitney, *p<0.05*. (**E**) 12-month-old WT females have larger structures than age-matched males. *Mann-Whitney, **p<0.01*.

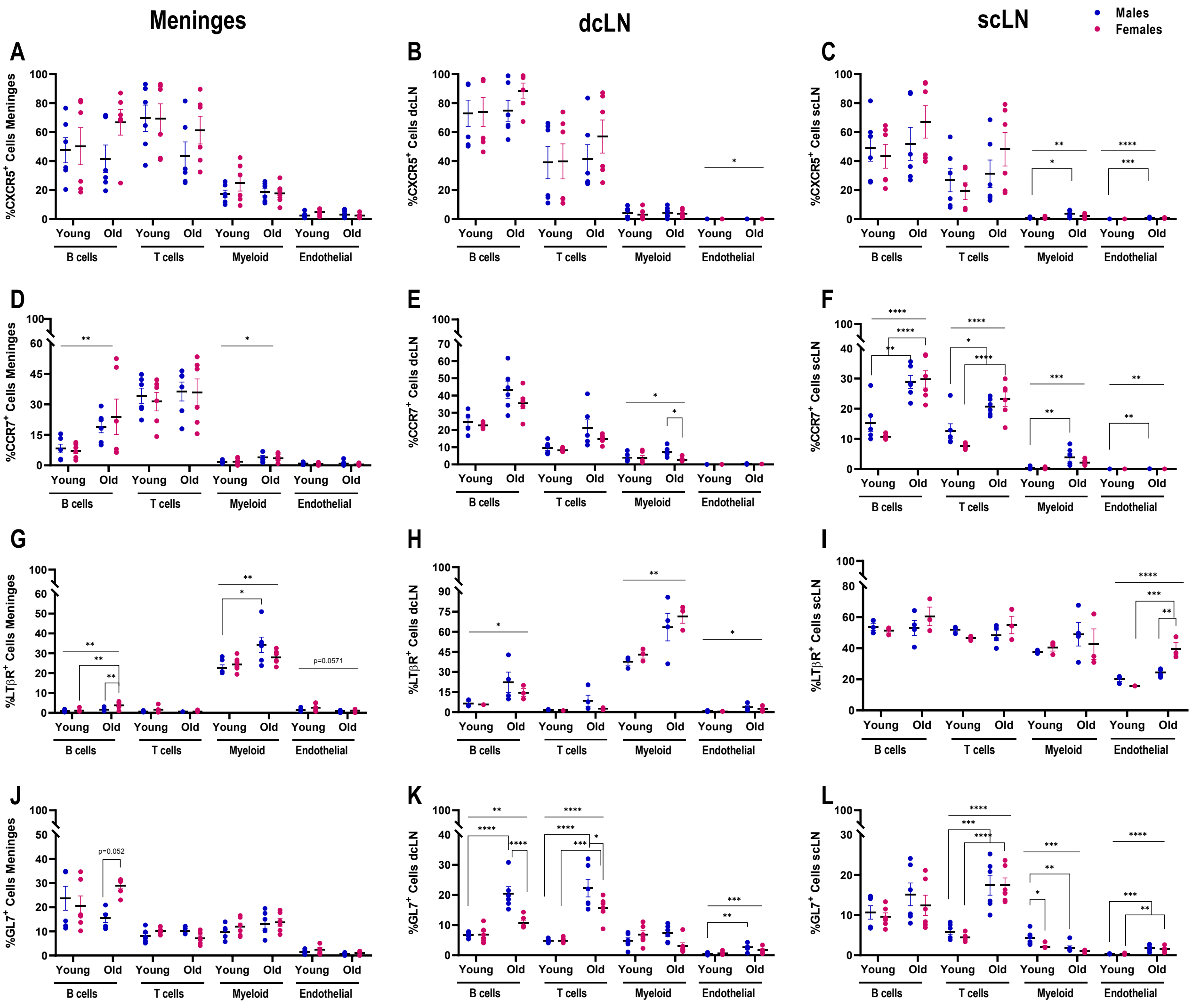

Figure S2. Flow cytometry analysis of immune cell populations from the meninges, dcLN, and scLN. The immune cell populations include CD45$^+$CD45R$^+$ B cells, CD45$^+$CD3$^+$ T cells, CD45$^+$CD11b$^+$ myeloid cells, and CD31$^+$ endothelial cells. The expression percentages of the following ELS-related markers are shown: CXCR5 (**A-C**), CCR7 (**D-F**), LTβR (**G-I**), GL7 (**J-L**). Data presented for two age groups: Young (1-3 months) and Old (18+ months), in both males and females, with N=6 per group. Statistical significance was determined by *Two-way ANOVA. *p<0.05*, ***p<0.01*, ****p<0.001*, *****p<0.0001.*

A

	5xFAD					
	Males			Females		
	1 month	12 months	18+ months	1 month	12 months	18+ months
Number of mice	7	5	5	8	8	3
Minimum ELS number	0	15	15	2	9	12
Maximum ELS number	7	36	72	4	44	37
Mean number of ELS per group	2.143	26.8	34.8	2.875	21.13	25
Std. Dev	2.545	8.319	23.53	0.991	11.75	12.53
SEM	0.9619	3.72	10.52	0.3504	4.155	7.234

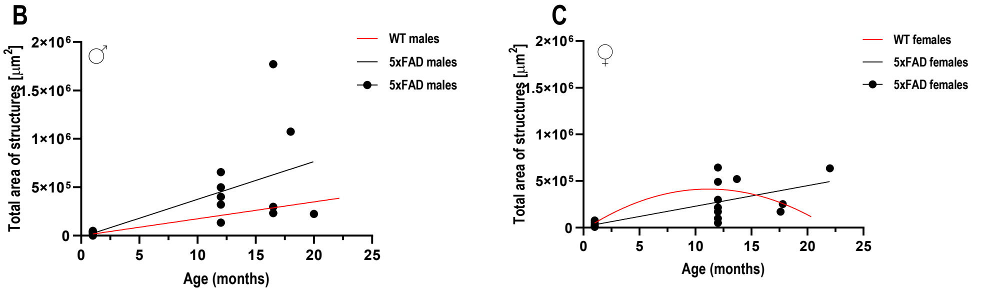

Figure S3. (**A**) Descriptive statistics for the number of ELS per group in the 5xFAD groups. (**B**) Correlation of total ELS area (mm^2) and age, linear regression in 5xFAD, and linear regression in age-matched WT (red) male mice. *Spearman's r=0.76, p<0.001* (**C**) Correlation of total ELS area (mm^2) and age in 5xFAD and non-linear fit regression in age-matched WT (red) female mice, *Spearman's r=0.80, p<0.0001*.

A

	APP/PS1					
	Males			Females		
	1 month	12 months	18+ months	1 month	12 months	18+ months
Number of mice	7	7	7	8	9	7
Minimum ELS number	8	5	3	6	0	3
Maximum ELS number	30	57	13	31	48	18
Mean number of ELS per group	17.29	16.57	8	14.63	22.78	10
Std. Dev	7.653	18.11	4.203	8.123	18.79	6
SEM	2.893	6.845	1.589	2.872	6.262	2.268

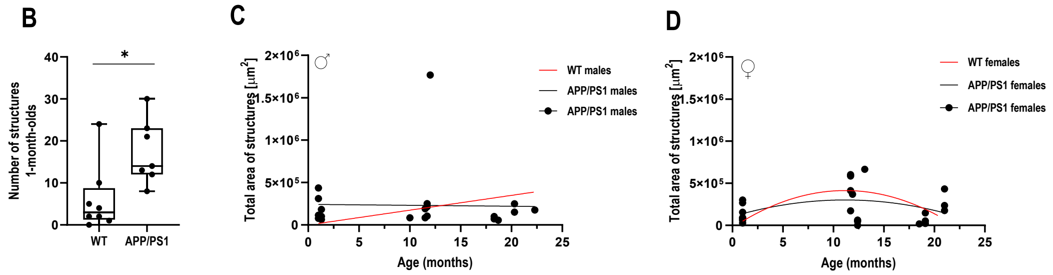

Figure S4. (**A**) Descriptive statistics for the number of ELS per group in the APP/PS1 groups. (**B**) Correlation of total ELS area (mm^2) and age in APP/PS1, and linear regression in age-matched WT (red) male mice. *Spearman's r=-0.0.11*, *p=ns* (**C**) Correlation of total ELS area (mm^2) and age in APP/PS1 and non-linear fit regression in age-matched WT (red) female mice, *Spearman's r=0, p=ns*. (**D**) 1-month-old APP/PS1 male mice have more dural ELS than age-matched WT, *Mann-Whitney *p<0.05*.

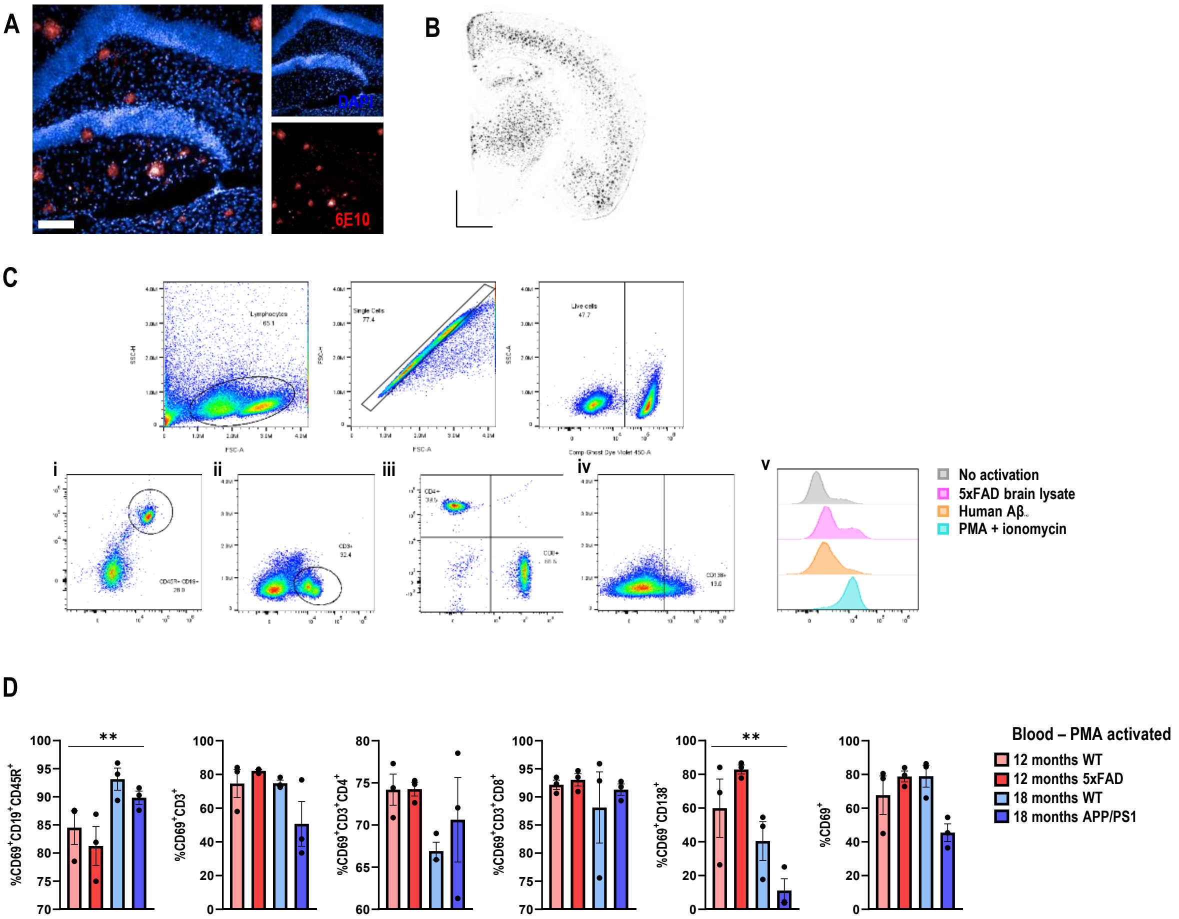

Figure S5. (**A**) Representation of hippocampal staining for Aβ plaques (6E10, red) and DAPI (blue). Scale bar 100 µm. (**B**) An example of a wholemount scan of a brain hippocampal section stained for 6E10. Greyscale. 1mm scale bar. (**C**) Flow cytometry gating exemplar. All samples were gated for live CD45+ cells. i CD19+CD45R+ B cells, ii CD3+ T cells, iii CD3+CD4+ T helper cells and CD3+CD8+ cytotoxic T cells, iv CD138+ plasma cells, v CD69+ activated cells. (**D**) Flow cytometric analysis of PMA-activated adaptive immune cell populations in the blood of either 12-month-old WT, 18-month-old WT, 12-month-old 5xFAD, and 18-month-old APP/PS1 males. *Kruskal-Wallis **p<0.01.*

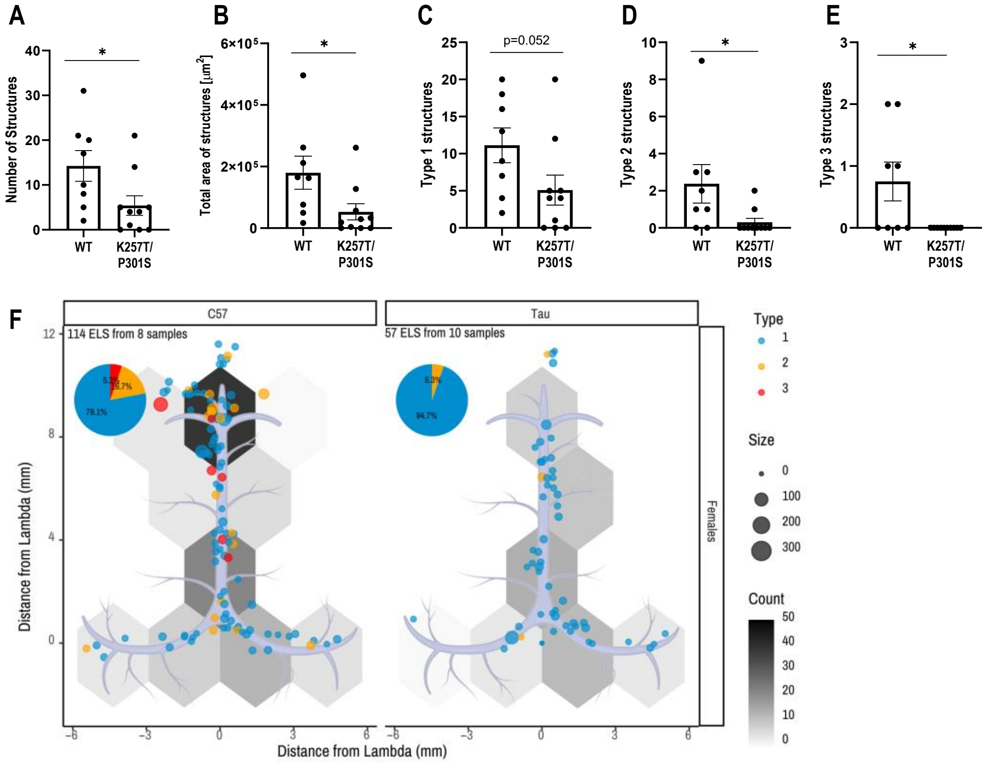

Figure S6. Mice with the K257T/P301S mutation in Tau exhibit reduced meningeal ELS formation. Meninges from female mice with the K257T/P301S Tau mutation (n=10) and WT mice (n=8) were analyzed for ELS formation. (**A**) The number of ELS in K257T/P301S is lower than in WT mice. (**B**) The total area of ELS in K257T/P301S is lower than in WT mice. (**C**) Number of type 1 ELS in K257/P301S is lower than in WT mice. (**D**) Number of type 2 ELS in K257T/P301S is lower than in WT mice. (**E**) Number of type 3 ELS in K257T/P301S is lower than in WT. (**F**) ELS density map in the dural sinuses of K257T/P301S female mice compared to age-matched WT females. *Mann-Whitney *p<0.05.*

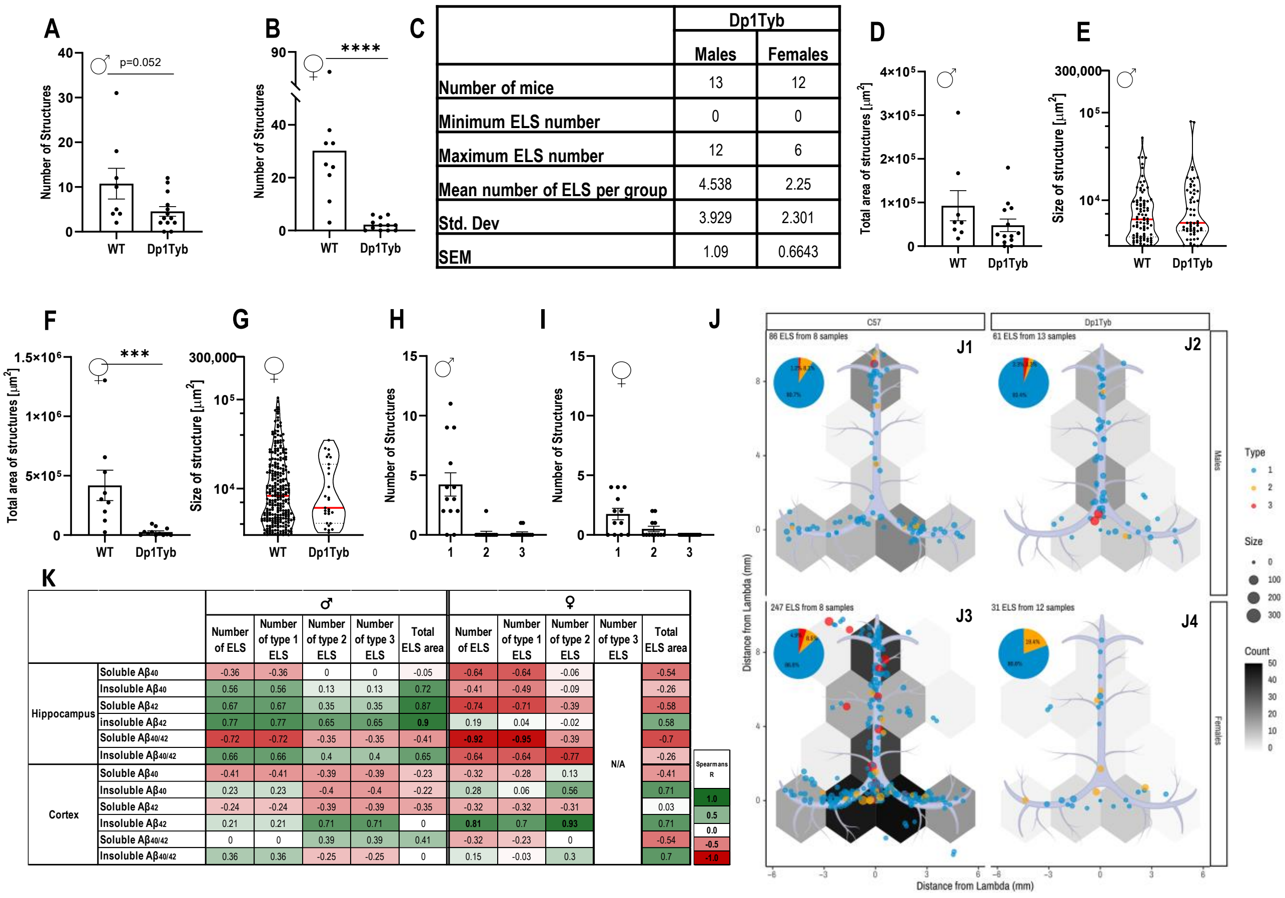

	Dp1Tyb	
	Males	Females
Number of mice	13	12
Minimum ELS number	0	0
Maximum ELS number	12	6
Mean number of ELS per group	4.538	2.25
Std. Dev	3.929	2.301
SEM	1.09	0.6643

		♂					♀				
		Number of ELS	Number of type 1 ELS	Number of type 2 ELS	Number of type 3 ELS	Total ELS area	Number of ELS	Number of type 1 ELS	Number of type 2 ELS	Number of type 3 ELS	Total ELS area
Hippocampus	Soluble Aβ40	-0.36	-0.36	0	0	-0.05	-0.64	-0.64	-0.06	N/A	-0.54
	Insoluble Aβ40	0.56	0.56	0.13	0.13	0.72	-0.41	-0.49	-0.09		-0.26
	Soluble Aβ42	0.67	0.67	0.35	0.35	0.87	-0.74	-0.71	-0.39		-0.58
	insoluble Aβ42	0.77	0.77	0.65	0.65	**0.9**	0.19	0.04	-0.02		0.58
	Soluble Aβ40/42	-0.72	-0.72	-0.35	-0.35	-0.41	**-0.92**	**-0.95**	-0.39		-0.7
	Insoluble Aβ40/42	0.66	0.66	0.4	0.4	0.65	-0.64	-0.64	-0.77		-0.26
Cortex	Soluble Aβ40	-0.41	-0.41	-0.39	-0.39	-0.23	-0.32	-0.28	0.13		-0.41
	Insoluble Aβ40	0.23	0.23	-0.4	-0.4	-0.22	0.28	0.06	0.56		0.71
	Soluble Aβ42	-0.24	-0.24	-0.39	-0.39	-0.35	-0.32	-0.32	-0.31		0.03
	Insoluble Aβ42	0.21	0.21	0.71	0.71	0	**0.81**	0.7	**0.93**		0.71
	Soluble Aβ40/42	0	0	0.39	0.39	0.41	-0.32	-0.23	0		-0.54
	Insoluble Aβ40/42	0.36	0.36	-0.25	-0.25	0	0.15	-0.03	0.3		0.7

Spearmans R
1.0
0.5
0.0
-0.5
-1.0

Figure S7. 10-15-months-old male and female Dp1Tyb mice exhibit reduced dural ELS formation. Dural wholemounts from 10-15-month-old male and female Dp1Tyb mice (n=12-13) were analyzed for the presence of ELS structures and compared to 12-month-old WT males and females accordingly (n=8-9). (**A**) Number of ELS of Dp1Tyb males compared to WT males. (**B**) Number of ELS structures of Dp1Tyb females compared to WT females. *Mann-Whitney, ****p<0.0001.* (**C**) Descriptive statistics for the number of ELS per group in the Dp1Tyb groups. (**D**) Total ELS area (mm^2) per meninges of Dp1Tyb males compared to WT males. (**E**) Violin plot of ELS size (μm^2) of all individual structures of Dp1Tyb males compared to WT males. The median is represented as a red line. (**F**) Total ELS area (mm^2) per meninges of Dp1Tyb females compared to WT females. *Mann-Whitney, ***p<0.001.* (**G**) Violin plot of ELS size (μm^2) of all individual structures of Dp1Tyb females compared to WT females. The median is represented as a red line. (**H-I**) Number of type 1, 2, and 3 ELS of (**H**) Dp1Tyb males and (**I**) Dp1Tyb females. (**J**) ELS Density map in the Dural sinuses of (**J$_1$**) WT males, (**J$_2$**) Dp1Tyb males, (**J$_3$**) WT females, and (**J$_4$**) Dp1Tyb females. (**K**) Correlations of parenchymal Aβ pathology (Aβ/total protein mass (μg/mg)) and ELS in 10-15-months-old Dp1Tyb mice (n=5-6). Spearman's R color scale is shown on the right. Colored values represent significant correlations.

Figure Video S1. 3D video of a type 3 lymphoid structure in the sagittal sinus. CD45R+ B cells (red), $CD3^{+}$ T cells (cyan), and $Ki67^{+}$ cells (yellow).

		ELS in healthy aging	ELS in AD-related neurodegeneration	ELS in autoimmune (MS and EAE) CNS Pathology[1,2]	ELS in Glioma[1,2]
**Effectors of meningeal ELS formation**	**Preclinical mouse models**	Not reported	ELS are not surrounding Aβ in 5xFAD, and peripheral immune cells are not activated by Aβ	Found in models immunized with self-antigens, and induced by Th17	CD40 ligand expression in murine brainstem tumors increases meningeal ELS formation
	**Human tissues**	Not reported	Not reported	CNS self-antigens, found at chronic stages of the disease	Relatively rare, TLSs are found mainly in WHO grade II-IV gliomas
**ELS function**	**Preclinical mouse models**	Not reported	Unknown	Local site for B cell expansion and plasma cell formation	Enhanced T cell infiltration, regulation of brain tumor immunity
	**Human tissues**	Not reported	Not reported		Enhanced T cell infiltration, including immunosuppressive cells in the structures[3]
**Disease outcome**	**Preclinical mouse models**	Not relevant	Correlates with more severe brain pathology in 5xFAD but not in APP/PS1	Higher number of lesions	Extended survival
	**Human tissues**	Not relevant	Not reported	Demyelinated regions	Inconclusive
**Germinal Center presence**	**Preclinical mouse models**	No type 3 ELS in young mice and increased type 3 ELS formation as age progresses.	Increased type 3 as age progresses, positively correlated to plaque load	Inconclusive for GC-like response in early EAE, while ELS development occurs as pathology progresses	Not reported
	**Human tissues**	Not reported	Not reported	Reported in secondary progressive MS patients[4] and early MS	Not reported
**Sex differences**	**Preclinical mouse models**	Increases with age in males, but is dynamic in females	No difference between males and females in 5xFAD, mild differences in APP/PS1	No differences indicated	No differences indicated
	**Human tissues**	Not reported	Not reported	No differences indicated	No differences indicated

Table S1. ELS comparison across different CNS pathologies. Relevant references appear in the manuscript.

References.

1. Yang, C., Cai, Y.-X., Wang, Z.-F., Tian, S.-F. & Li, Z.-Q. Tertiary lymphoid structures in the central nervous system. *Trends in Molecular Medicine* **0**, (2024).
2. van de Walle, T. *et al.* Tertiary Lymphoid Structures in the Central Nervous System: Implications for Glioblastoma. *Front. Immunol.* **12**, 724739 (2021).
3. Chen, J., Yang, Y., Luan, S., Xu, W. & Gao, Y. Tertiary lymphoid structures in gliomas: impact on tumour immunity and progression. *Journal of Translational Medicine* **23**, 528 (2025).
4. Serafini, B., Rosicarelli, B., Magliozzi, R., Stigliano, E. & Aloisi, F. Detection of Ectopic B-cell Follicles with Germinal Centers in the Meninges of Patients with Secondary Progressive Multiple Sclerosis. *Brain Pathology* **14**, 164–174 (2004).

Methods.

Animals. All mice were group housed in the Bar-Ilan University specific pathogen-free (SPF) animal facility at 22°C and a 12h light/dark cycle with access to ad-libitum food and water. Animal care, genotyping, and experiments were approved by local ethical committees. Male and Female C57bl/6 wild-type mice (Harlan, Israel) were used as control mice. The 5xFAD mouse model of early-onset AD (EOAD) (strain #34840, Jackson Laboratories, Bar Harbor, ME, USA) expresses human APP and PS1 transgenes with a total of five AD-linked mutations: the Swedish (K670N/M671L), Florida (I716V), and London (V717I) mutations in APP, and the M146L and L286V mutations in PS1. The APP/PS1 mouse strain (C57BL/6J-TgN Thy1-APPK670M/671NL; Thy1-PS1L166P), which also models EOAD, expresses the Swedish mutation APPKM670/671NL and the presenilin 1 mutation, PS1I166P, and was provided by Prof. Mathias Jucker of the German Center for Neurodegenerative Diseases, Tubingen, Germany. Both these strains exhibit accelerated Aβ deposition, behavioral deficits, and neuronal loss, hence modeling EOAD (Oakley et al., 2006; Sasaguri et al., 2017). To model Tau hyperphosphorylation, a mouse model with the double mutation (DM) in the human Tau protein (K257T/P301S)[66] was used. Dp1Tyb strain (provided by Dr. Frances Wiseman from the UK Dementia Research Institute, London, UK) was used to model APP-related pathology in DS. These carry a Mmu16 duplication between the Lipi and Zbtb21 genes, containing 63% of the Human chromosome 21 orthologous genes, including a significantly higher expression of the APP and Dyrk1a genes[31]. The number of mice used for each experiment is indicated in the relevant figure legend.

Immunofluorescence analysis of meningeal wholemounts. Mice were perfused with ice-cold PBS to analyze meningeal wholemounts using IF. Skulls were carefully collected and preserved with 2% PFA for subsequent processing. Prior to staining, skulls were washed with PBS, and the dura was gently peeled from the skull bone. The tissues were then washed with

PBS-Triton 0.1% and blocked for 1 hr (10% NHS, 2% BSA, 1% Glycine in PBS-Triton 0.1%) before being incubated with the primary anti-CD3 (1:400, Abcam, ab33429) and anti-Ki67 (1:500, Abcam, ab15580) monoclonal antibodies for 3 hrs at room temperature (RT). Following the primary antibody incubation, the tissues underwent 3 washes with PBS-Triton 0.1% for 5 min each. Subsequently, the tissues were incubated with secondary antibodies Alexa Fluor® 647 goat anti-rat (1:1000, Rhenium) and anti-rabbit Alexa Fluor® 488 (1:1000, Rhenium) for 1 hr, followed by additional 3 washes with PBS-Triton 0.1% for 5 min each. Next, the meninges were incubated overnight with a conjugated (PE-CF594) rat anti-mouse CD45R monoclonal antibody (562290, BD Horizon™). After 3 additional washes with PBS-Triton 0.1%, the meninges were stained with nucleic acid stain (Hoechst 33342, ThermoFisher Scientific) for 10 min at RT. Finally, the meninges were washed three times with PBS and mounted on positively charged slides for imaging. Slides were scanned using The Opera Phenix® Plus High-Content Screening Confocal System by Revvity using X20 water objective, with 11 Z-sections over a total of 16μm depth for meningeal tissue slides.

Meningeal wholemount image analysis was performed using FIJI (ImageJ) software (version 1.54f, National Institutes of Health, USA)[77]. A custom macro was developed to automate the analysis process. For each image, the macro located dense cell structures and computed a measurement of the area for each detected structure. Artifacts and small aggregations (less than 3000μm^2) were excluded. Data was then exported to a spreadsheet, and each structure was manually classified into one of three morphology types of ectopic lymphoid structures. Investigators were blinded to the group assignment of each sample.

For meningeal Aβ staining, the meningeal tissue of a 20-month-old 5xFAD male was processed as described before. Followed with a primary antibody staining for anti-Amyloid-beta (1:500 6E10-Alexa Fluor® 647, Biolegend, cat. 803001), conjugated (PE-CF594) rat anti-mouse CD45R monoclonal antibody (1:200, 562290, BD Horizon™) and anti-CD3 (1:400, Abcam,

ab33429) overnight at 4ºC. Then, it was washed and stained with secondary goat anti-rat Alexa Fluor® 488 for 1 hr RT, followed by the same steps previously mentioned for other meningeal staining.

Amyloid plaque analysis. For plaque analysis, one hemisphere of 5xFAD and APP/PS1 brains was collected to 4% PFA following perfusion. Hemi-brains were transferred to 20% glucose overnight, then 30% overnight. Forty microns of Cryostat sections containing the hippocampus were collected. Sections were washed in PBS-Triton 0.1% 5 min 3 times, then blocked (as mentioned before) for 1 hr at RT and incubated overnight with anti-Amyloid-beta (6E10 #SIG-39320 conjugated Alexa Fluor® 647). Then washed and stained with the nucleic acid stain (Hoechst 33342, ThermoFisher Scientific) for 10 min at RT. Finally, the sections were washed three times with PBS and mounted on positively charged slides for imaging. Slides were scanned using The Opera Phenix® Plus High-Content Screening Confocal System by Revvity using X20 water objective, with 4 Z-sections over a total of 11µm depth for brain section slides.

Brain wholemount image analysis was performed using FIJI (ImageJ) software (version 1.54f, National Institutes of Health, USA)[77]. A custom macro was developed to automate the analysis process. For each image, the macro located stained regions of the antibody and measured the size and quantity, along with the size of the whole section. Artifacts and small plaques under $100\mu m^2$ were excluded.

Meningeal ELS density map. For the density map analysis, a reproducible R pipeline was implemented in the R statistical language v4.4 (R Core Team, 2024). _R: A Language and Environment for Statistical Computing_. R Foundation for Statistical Computing, Vienna, Austria. Results were visualized with the ggplot2 v3.5.1 (H. Wickham. ggplot2: Elegant Graphics for Data Analysis. Springer-Verlag New York, 2016.), and patchwork v1.2.0 packages (Pedersen T (2024). _patchwork: The Composer of Plots_. R package version 1.2.0.).

The analytical scripts are available at https://doi.org/10.5281/zenodo.15453825 under an Artistic 2.0 license[78].

Flow Cytometry. Blood was collected from the facial vein, followed by PBMC isolation using 5 mL ACK lysis for 5 min. To stop the red blood cell lysis, 8 mL of complete culture medium (2 mM L-glutamine, 0.1 mM penicillin-streptomycin-nystatin solution, 0.1 mM 2-mercaptoethanol, 10% FBS in RPMI) was added. Mice were sacrificed and perfused with ice-cold PBS. Superficial and deep cervical lymph nodes were collected and forced through a cell-strainer cap (Fisher Scientific, #352235) into a 5 mL tube. The dural meninges were immediately collected in 0.5 ml RPMI, then transferred to lysis buffer containing 2% FBS, 1mg/ml collagenase VIII, and 0.5mg/ml DNase I in RPMI, for 25 min at 37°C. All cell suspensions were pelleted (400 x g, 5 minutes, 4 °C). For WBC activation, blood and dcLN cells were resuspended in 1 mL culture medium and transferred to a 24-well culture plate. For PBMC activation, one of the following was added to the proper wells: (i) 50 ng/mL PMA and 500 ng/mL ionomycin, (ii) brain lysate containing 200 µg of total protein, (iii) 10 µg/mL synthetic human $A\beta 1_{42}$. For dcLN activation, 10 µg synthetic human $A\beta 1_{42}$ per sample was added following oligomerization by diluting it to a final concentration of 100 µM in PBS and incubating at 4°C for 24 hours. Cells were incubated in a 5% CO_2 incubator at 37°C for 24 hours (with either brain lysate, human $A\beta 1_{42}$, or non-activated samples). Following incubation, the cells were pulled down for subsequent staining. All tissue pellets were stained with either Ghost Dye Violet 450 or Ghost Dye 780 (1:1000) for 30 minutes at 4°C, followed by blocking in 3% BSA-PBS for 15 minutes. Activation cohort samples were stained 30 min with CD69-FITC (1:100, 104505, BioLegend), CD45R-VioGreen (1:200, 130-110-852, Miltenyi Biotec), CD19-PE-Cy7 (1:200, 552854, BD Pharmingen™), CD138-BV711 (1:100, 563193, BD Biosciences), CD3-AF532 (1:100, 58-0032-82, Thermo Fisher), CD4-PE (1:500, 100408, BioLegend), CD8-APC (1:500, 100712, BioLegend). The rest of the samples were stained with

CD45R-VioGreen (1:200, 130-110-852, Miltenyi Biotec), CD3-AF532 (1:100, 58-0032-82, Thermo Fisher), CD45 (1:1000, 103127, BioLegend), CD11b (1:500, 25-0112-82, Invitrogen), CD31-BV421 (1:500, 563356, BD Horizon™), GL7-Alexa Fluor® 488 (1:1000, 144611, BioLegend), BCL6-APC (1:500, 130-118-346, Miltenyi biotec), LTβR-PE (1:200, 130-106-636 Miltenyi biotec), CXCR5(CD185)-PEvio615 (1:500, 130-107-656 Miltenyi biotec), CCR7(CD197)-BV650 (1:200, 134403, BioLegend). All antibodies were titrated prior to use. Data were recorded on a Cytek® Aurora spectral flow cytometer. An equal number of live cells was exported from each sample and analyzed in FlowJo software v10.10.0.

Brain Lysate preparation for immune cell activation assays. Mouse brains were collected and stored at -8 °C and then homogenized using a dry ice-assisted homogenizer, with two 15-second grinding and 30-second rest cycles at 5000 rpm, followed by four additional sets of four cycles at 6000 rpm. Lysates were transferred to Eppendorf tubes and centrifuged four times at 20000xg for 20 min at 4°C. Supernatants were snap-frozen on dry ice. Total protein concentration was determined using a BCA assay and normalized between samples.

Quantitative real-time PCR (RT-PCR). Following animal sacrifice (described above), total cellular RNA was extracted from homogenized tissues using TRIzol reagent (Invitrogen) following the manufacturer's protocol. cDNA was prepared using RevertAid H Minus First Strand cDNA Synthesis Kit (Thermo Scientific, Cat. K1631), and quantitative RT-PCR was performed using the Fast SYBR™ Green Master Mix (Applied Biosystems™, Cat. 4385612) on a StepOnePlus Real-Time PCR Systems (Applied Biosystems™). Expression levels were measured using the following primers: actin, fwd 5'- -3', rvs 5'- -3'; LTβ, fwd 5'-CTGCCCACCTCATAGGCGCTTG-3', rvs 5'-GACGCCGTCCTGTGGCAGCG-3'; LTβR, fwd 5'-CCCATACCAGATGTGAGATCC-3', rvs 5'-GGTGAAGAGCAGAAAGAGGAC-3'; CXCL13, fwd 5'-TCTCTCCAGGCCACGGTATT-3', rvs 5'-GGGGCGTAACTTGAATCCGA-3'; TNFα, fwd 5'-TGCCTATGTCTCAGCCTCTTC-3',

rvs 5'-GAGGCCATTTGGGAACTTCT-3'. Fold Change ratio was computed using the pfaffl method[79].

Measuring mouse Aβ levels using sandwich-ELISA. 96-well microplates (655061, Greiner) were covered with either anti-$A\beta_{42}$ antibody 12F4 (1:100, Biolegend) for mouse $A\beta_{42}$ measurement and anti-$A\beta_{40}$ antibody EPR23712-2 for $A\beta_{40}$ measurement (1:1000, ab254345, abcam), diluted in carbonate-bicarbonate buffer (pH = 9.6) and incubated overnight at 4ºC. Plates were washed 4 times in PBS-Tween solution (0.05% Tween in PBS) and blocked with 2% BSA solution in PBS-Tween for 1 hr at RT. Following 4 washes with PBS-Tween, tissue lysates were applied to each well and incubated for 2 hr at RT. Plates were then washed 5 times in PBS-Tween, and the detection HRP anti-Aβ, 17-24 antibody 4G8 (1:750 for $A\beta_{42}$, 1:500 for $A\beta_{40}$, Biolegend) was added and incubated for 2 hr at RT. Later, plates were washed 5 times in PBS-Tween and covered with 3,3',5',5'-tetramethylbenzidine (TMB) substrate (Cat# 00-4201-56, eBioscience Thermo Fisher). The color reaction was stopped by adding H_2SO_4. Optical density (OD) was measured at 450 nm using a spectrophotometer. Levels of $A\beta_{42}$ and $A\beta_{40}$ are indicated as the amount of protein out of total protein mass (µg/mg).

Statistics. A post hoc power analysis was performed using G*Power 3.1 software to evaluate the achieved power of the study based on the sample size and observed effect size. The analysis utilized the following parameters: a sample size of 16 participants (8 per group), a significance level of $\alpha = 0.05$, and the observed effect size (Cohen's d) = 1.953 derived from the study results. Resulting in a power of 0.952. To assess differences in the number and size of ELS, a correlation coefficient was computed for several structures and ages, and a simple linear regression was computed (a non-linear regression line was computed for C57 females due to different data dynamics). Where indicated, data are presented as mean±SEM. Where indicated, data were analyzed using either one-way ANOVA, two-way ANOVA, multiple *t-tests*, or the student's *t-test* (and related non-parametric tests for non-normally distributed data), while

adjusting the *p-value* for multiple comparisons using Holm-Sidak. Spearman's correlation was used for plaque and Aβ correlations shown in Figures 5 and 7. Statistical tests were performed using GraphPad Prism version 8.4.2 for Windows, GraphPad Software, Boston, Massachusetts, USA, www.graphpad.com. Outliers were intentionally not excluded to depict the genuine characteristics of the phenotypes. Significant results are marked according to conventional critical *p-values*: $^{*}p<0.05$, $^{**}p<0.01$, $^{***}p<0.001$, $^{****}p<0.0001$.
